# The bioelectric mechanisms of local calcium dynamics in cancer cell proliferation: an extension of the A549 *in silico* cell model

**DOI:** 10.3389/fmolb.2024.1394398

**Published:** 2024-05-06

**Authors:** Sonja Langthaler, Christian Zumpf, Theresa Rienmüller, Niroj Shrestha, Julia Fuchs, Rui Zhou, Brigitte Pelzmann, Klaus Zorn-Pauly, Eleonore Fröhlich, Seth H. Weinberg, Christian Baumgartner

**Affiliations:** ^1^ Institute of Health Care Engineering with European Testing Center for Medical Devices, Graz University of Technology, Graz, Austria; ^2^ Gottfried Schatz Research Center for Cell Signaling, Metabolism and Aging, Medical University of Graz, Graz, Austria; ^3^ Research Unit on Cell Biology, Histology and Embryology, Gottfried Schatz Research Center for Cell Signaling, Metabolism and Aging, Medical University of Graz, Graz, Austria; ^4^ Center for Medical Research, Medical University of Graz, Graz, Austria; ^5^ Department of Biomedical Engineering, The Ohio State University, Columbus, OH, United States; ^6^ Davis Heart and Lung Research Institute, The Ohio State University Wexner Medical Center, Columbus, OH, United States

**Keywords:** *in silico* models, electrophysiology, calcium dynamics, A549 cell line, cancer

## Abstract

**Introduction:**

Advances in molecular targeting of ion channels may open up new avenues for therapeutic approaches in cancer based on the cells’ bioelectric properties. In addition to *in-vitro* or *in-vivo* models, *in silico* models can provide deeper insight into the complex role of electrophysiology in cancer and reveal the impact of altered ion channel expression and the membrane potential on malignant processes. The A549 *in silico* model is the first computational cancer whole-cell ion current model that simulates the bioelectric mechanisms of the human non-small cell lung cancer cell line A549 during the different phases of the cell cycle. This work extends the existing model with a detailed mathematical description of the store-operated Ca^2+^ entry (SOCE) and the complex local intracellular calcium dynamics, which significantly affect the entire electrophysiological properties of the cell and regulate cell cycle progression.

**Methods:**

The initial model was extended by a multicompartmental approach, addressing the heterogenous calcium profile and dynamics in the ER-PM junction provoked by local calcium entry of store-operated calcium channels (SOCs) and uptake by SERCA pumps. Changes of cytosolic calcium levels due to diffusion from the ER-PM junction, release from the ER by RyR channels and IP3 receptors, as well as corresponding PM channels were simulated and the dynamics evaluated based on calcium imaging data. The model parameters were fitted to available data from two published experimental studies, showing the function of CRAC channels and indirectly of IP3R, RyR and PMCA via changes of the cytosolic calcium levels.

**Results:**

The proposed calcium description accurately reproduces the dynamics of calcium imaging data and simulates the SOCE mechanisms. In addition, simulations of the combined A549-SOCE model in distinct phases of the cell cycle demonstrate how Ca^2+^ - dynamics influence responding channels such as KCa, and consequently modulate the membrane potential accordingly.

**Discussion:**

Local calcium distribution and time evolution in microdomains of the cell significantly impact the overall electrophysiological properties and exert control over cell cycle progression. By providing a more profound description, the extended A549-SOCE model represents an important step on the route towards a valid model for oncological research and *in silico* supported development of novel therapeutic strategies.

## 1 Introduction

Ion transport across the cell and intracellular membranes through channels and carriers is involved in the regulation of survival, proliferation, death, and motility of cancer cells. Ca^2+^ ions play a fundamental role in a variety of intracellular signaling pathways that regulate cell proliferation, metabolism, excitability and contractability, exocytosis, gene transcription and expression. At rest, the cytosolic Ca^2+^ concentration [Ca^2+^]_Cyt_ is typically maintained low at around 100 nM, compared with the extracellular space with [Ca^2+^]_Ext_ > 1 mM and intracellular Ca^2+^ stores, such as the endoplasmic reticulum (ER) with [Ca^2+^]_ER_ > 100 µM (Bong and Monteith, 2018). A significant portion of intracellular Ca^2+^ is not free but is bound to numerous calcium binding proteins that can function as Ca^2+^ sensors, Ca^2+^ buffers or both. Some of the important Ca^2+^ buffers include calmodulin (CaM) in the cytosol, and calsequestrin (CSQ) and calreticulin in the ER (Islam, 2020).

In the context of the cell cycle, Ca^2+^ is crucial for regulating important steps such as DNA synthesis, cell cycle checkpoints, and phase transitions. During the G1 phase, for example, it modulates the transcription of immediate early genes necessary for cell proliferation and activates the cyclin-dependent protein kinases (CDKs) in association with their regulators, the cyclins, which, after a series of intermediate reactions, allow the transition from cell cycle phase G1 to S (Humeau et al., 2018). Therefore, maintaining balanced Ca^2+^ levels within intracellular compartments is crucial for the proper cell function and life cycle (Roderick and Cook, 2008). To enable controlled Ca^2+^ entry and release, each cell has a unique expression pattern of calcium regulating agents that determine its calcium homeostasis (Islam, 2020). The main players in calcium signaling include ion channels, which act as gatekeepers for Ca^2+^ entry into cells and regulate calcium exchange in different compartments. While voltage-gated calcium channels are a characteristic feature of excitable cells, ligand-gated calcium channels occur predominantly in non-excitable cells (Kozak and Putney, 2018). Store-operated calcium entry (SOCE) is one of the most important calcium entry pathways in non-excitable cells, and shows distinct behavior throughout the cell cycle. Exemplarily it is upregulated in G1/S transition and downregulated from S to G2/M phase (Chen et al., 2016; Hodeify et al., 2018). Although the molecular mechanism is not yet fully understood in detail (Islam, 2020), Ca^2+^ depletion from the endoplasmic reticulum (ER), induced by inositol 1,4,5 trisphosphate receptor (IP3R) and ryanodine receptor (RyR), triggers the SOCE mechanism to replenish the ER with Ca^2+^ and maintain Ca^2+^ signaling in the cytoplasm (Hogan, 2015). The two major SOCE proteins are the calcium-sensitive Stromal Interaction Molecule 1 (STIM1) on the ER membrane and the ORAI1 channel pore on the plasma membrane (PM) (Zheng et al., 2015), which together form the calcium release activated calcium (CRAC) channels that regulate Ca^2+^ influx across the PM into the cytosol. Incoming Ca^2+^ via CRAC channels binds to calmodulin to regulate downstream expression of cytokines, transcription factors, and enzymes, which in turn govern proliferation, differentiation, metabolism, mast cell degranulation or cytotoxicity (Hodeify et al., 2018; Vaeth and Feske, 2018). Other imported channels in Ca^2+^ exchange are the sarco/endoplasmic reticulum Ca^2+^ ATPase (SERCA) and the plasma membrane Ca^2+^ ATPase (PMCA), which pump Ca^2+^ from the cytoplasm into the ER and the extracellular space respectively (Liu, 2012; Liu, 2012).

Cancer cells exhibit altered ion channel expression or activity, which is closely linked to tumor development and progression (Kunzelmann, 2005; Schönherr, 2005; Fiske et al., 2006; Litan and Langhans, 2015; Brackenbury, 2016; Leanza et al., 2016). In particular, calcium channels involving ORAI and STIM, transient receptor potential (TRP) channels, as well as voltage-gated calcium (CaV) channels are reported to promote proliferation in various cancer types, for example the upregulated expression of ORAI3 and TRPV6 channels are known to enhance proliferation rates in prostate or breast cancer (Pinto et al., 2015; Dubois et al., 2014; Lehen’Kyi et al., 2007). Furthermore, cancer hallmarks such as self-sufficiency in growth signals is highly associated with calcium signaling and upregulation of various calcium channels, whereas reduction of calcium influx by downregulation of calcium channels has been shown to result in insensitivity to antigrowth signals and some resistance to apoptosis (Brackenbury, 2016; Lehen’Kyi et al., 2007; Prevarskaya et al., 2010; Raphaël et al., 2014; Thebault et al., 2006). Changes in ion channel expression consequently affect the membrane potential of the cell, which represents an important bioelectric signal for cell proliferation. In general, proliferating cells often have a more depolarized membrane potential V_m_ than quiescent cells. As reported in several studies, cells can be induced to enter or exit the cell cycle by external hyper- or depolarization of the membrane, respectively. For example, lowering the membrane potential of proliferating CHO cells has been shown to induce mitotic blockade, while depolarization of quiescent CHO cells can trigger mitosis. Similarly, sustained depolarization initiates DNA synthesis and mitosis in mature neurons, emphasizing V_m_ as a crucial regulator for the mitotic ability of cells (Cone and Tongier, 1971; Stillwell et al., 1973; Cone and Cone, 1976; Yang and Brackenbury, 2013; Rao et al., 2015). In fact, compared with normal cells, cancer cells typically tend to be more depolarized, which is assumed to trigger DNA-synthesis and mitosis, leading to increased proliferation. Furthermore, during cell cycle progression, the membrane potential undergoes rhythmic oscillations from hyperpolarized to depolarized states, which can trigger the transition and drive cells through the different cell cycle phases, thus serving as a putative bioelectric signal for cell proliferation (Yang and Brackenbury, 2013).

Recent advances in the field of cancer research have focused on targeting specific ion channels involved in tumor development and progression, which opens up promising therapeutic opportunities based on the unique electric properties of cancer cells. However, the intricate and multifaceted nature of the underlying mechanisms, ion channel activation and interactions, and its consequences for membrane potential necessitates a comprehensive and holistic perspective to effectively identify and explore potential key targets. To gain deeper insights into the complex relationship between electrophysiological properties and cancer, *in silico* models can be a valuable tool to study and understand the impact of altered ion channel expression and abnormal changes in membrane potentials on malignant processes (Langthaler et al., 2021; Baumgartner, 2022). In 2021 we developed the first *in silico* cell model in cancer electrophysiology using A549 human lung adenocarcinoma cells (Langthaler et al., 2021). This first hidden Markov-based model simulates the rhythmic oscillation of the membrane potential during the cell cycle, allowing a deeper understanding of the electrophysiological mechanisms during cell proliferation. Currently, the model incorporates 11 functionally expressed ion channels of the PM with the respective calcium and voltage dependencies of the individual ion channels. As far as calcium modeling is concerned, the estimation of the calcium concentration in the initial model is based on a simplified approach that assumes a certain steady-state concentration of the cytosol, without taking into account the complex local calcium dynamics within the cell. However, the local calcium distribution and time evolution in the different compartments of the cell are of great importance for the activity of certain types of ion channels and subsequently affect the overall electrical properties of the cell membrane.

In this work, building upon the initial model, we propose an extension that takes into account the complex dynamics of intracellular calcium. For this purpose, the initial model is extended by a multicompartmental approach that addresses the heterogenous calcium profile and dynamics in the ER-PM junction caused by local Ca^2+^ entry via SOCE and uptake by SERCA pumps. Changes in cytosolic calcium levels due to diffusion from the ER-PM junction, release from the ER by RyR channels and IP3 receptors, and responding PM channels are implemented and the dynamics are assessed using literature and experimental data published elsewhere. The aim of this extension is to establish a comprehensive framework that elucidates the complex interplay between ion channels, intracellular calcium dynamics and electrophysiological processes in non-excitable cancer cells in order to gain a deeper understanding of cell proliferation mechanisms and potentially contribute to the discovery of new therapeutic strategies for cancer treatment.

## 2 Materials and Methods

The initial A549 *in silico* model involves three ligand-gated Ca^2+^ channels of the plasma membrane, the CRACM1, TRPV3 and TRPC6 (Langthaler et al., 2021). These channels enable the Ca^2+^ influx from the extracellular space into the cytosol. However, there are additional important channels and ion pumps including the sarco/endoplasmic reticulum Ca^2+^ ATPase (SERCA), which pumps Ca^2+^ ions from the cytosol into the ER, the plasma membrane Ca^2+^ ATPase (PMCA), which pumps Ca^2+^ out of the cell to the extracellular space, the inositol 1,4,5 trisphosphate receptor (IP3R) and the ryanodine receptor (RyR), both releasing Ca^2+^ from the ER into the cytosol (Liu, 2012), which have not yet been considered in the model. A summary of reported ion channels in the human lung cancer cell line A549, the role and function of the individual ion channels and their impact on electrophysiological properties, and relevance on cell proliferation and tumor progression in A549 cells are provided in the Supplementary Material Part A.

There is a large variety of published models that describe the store-operated calcium entry (SOCE) and calcium dynamics of the ER-PM junction (Kowalewski et al., 2006; Means et al., 2006; Marhl et al., 2008; Liu, 2012; Schmeitz et al., 2013; Eichinger et al., 2018; McIvor et al., 2018), all following the same basic principle of the law of mass balance. The Ca^2+^-submodel added to the A549 cell model is largely based on the SOCE model described in Liu, 2012.

### 2.1 The SOCE model

**FIGURE 1:**
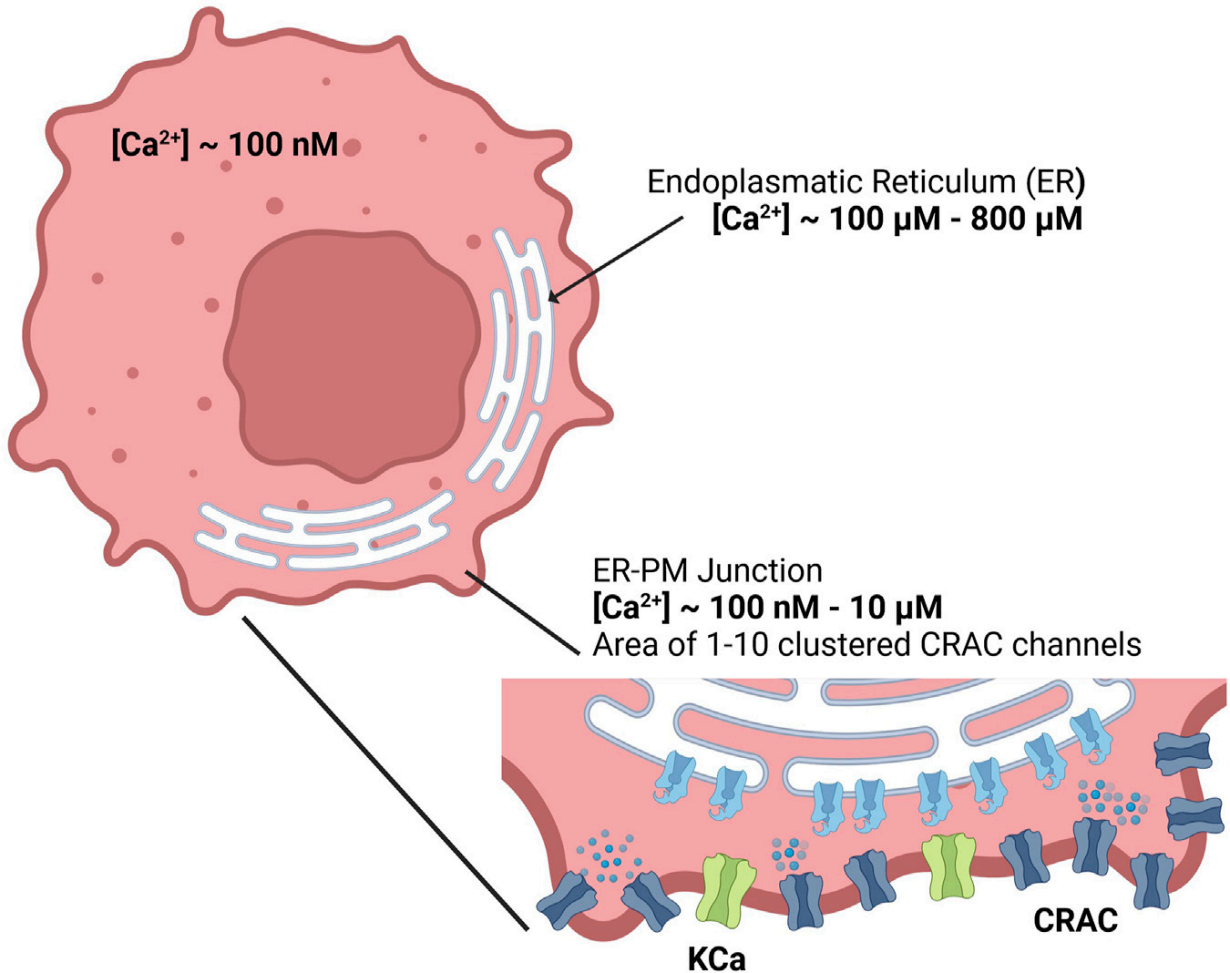
Calcium concentrations of the cytosol, endoplasmic reticulum and the ER-PM junction inside the cell.

**FIGURE 2:**
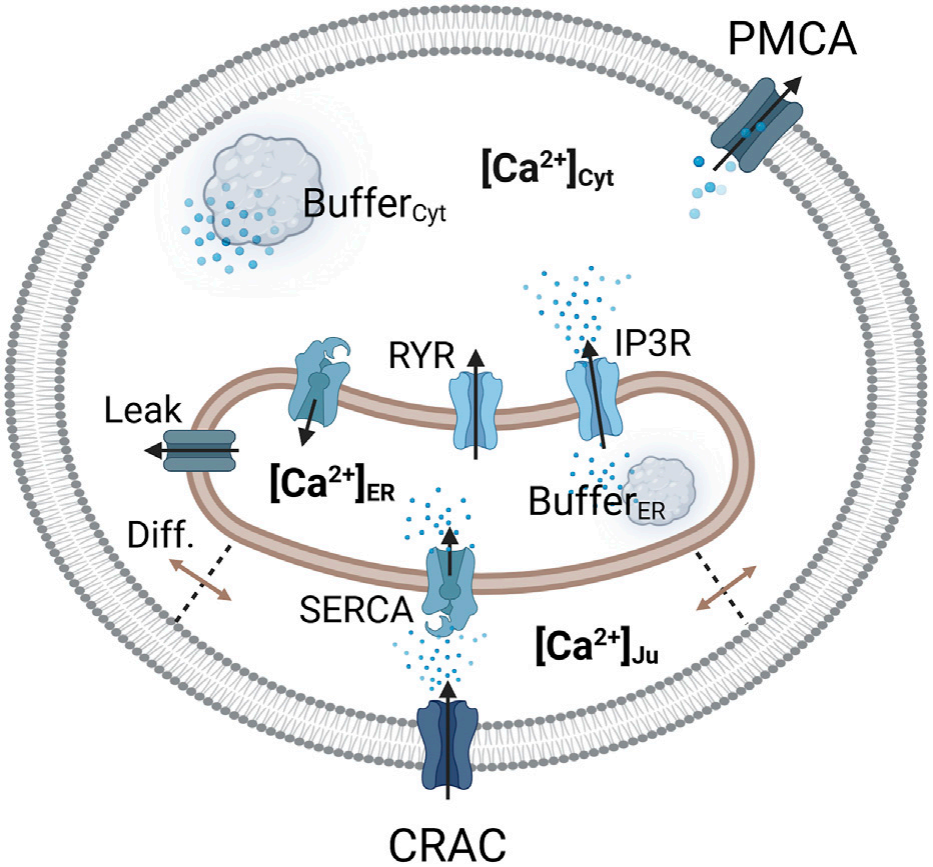
Visualization of the considered Ca^2+^ compartments and their connections in the SOCE model concept. The CRAC channel is formed by the ORAI1 channel on the PM and STIM1 on the ER membrane.

A depiction of the cell and corresponding calcium levels is given in Figure 1. The gating of the CRAC channels and to some extent the pump rate of the SERCA pumps depend on the calcium concentration inside the ER (Liu et al., 2010). The ER in turn is influenced by calcium entry through SERCA pumps, with which the cytosolic calcium concentration changes dynamically and which thus needs a separate description (Schmeitz et al., 2013). Taking the ER-PM junction into account, this results in three necessary calcium domains for a reliable model description. However, a real cell also contains additional important calcium pools such as the mitochondria or lysosomes, which are not considered at this level of abstraction. Additionally, the CRAC current in the SOCE model is assumed to be the only calcium influx into the cell, which however leaves out other calcium channels such as the TRP channels. The calcium pools are designed by the law of mass balance (Liu, 2012) which defines them by the sum of the calcium in- and outflows. All included calcium pools and flows as considered in this modeling approach are summarized in Figure 2.

Calcium enters the cytosol by diffusion from the junction, from the ER via the RyR and IP3R channels, and through an ER calcium leakage. The cytosol is cleared of calcium via the PMCA and the SERCA pumps, which are located in the plasma and ER membrane, respectively. The ER is filled with calcium via SERCA pumps, which are active in the junction and the cytosol, depending on the dynamics of local calcium concentrations. Therefore, two separate SERCA flows for the junction and the cytosol were implemented. The outflow from the ER is facilitated by IP3R channels, RyR channels and the ER leak. The majority of intracellular calcium is bound to calcium storage or buffer proteins (Liu et al., 2010; Islam, 2020). To account for buffered calcium, buffer terms were included for the cytosol and the ER, but not for the junction.

#### 2.1.1 Calcium dynamics in the ER-PM junction

**FIGURE 3:**
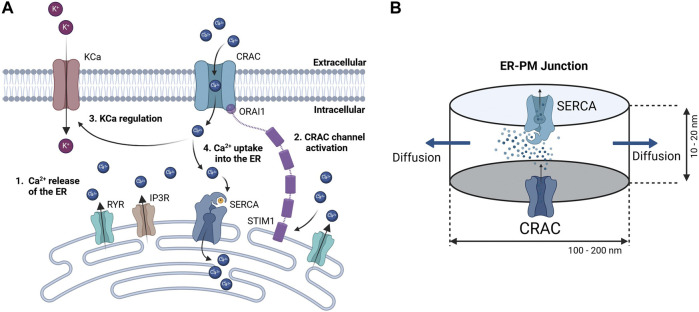
Concept of calcium dynamics in the **(A)** ER-PM junction and **(B)** single junction geometry and calcium flows for modeling. The junction is assumed to be a cylinder with CRAC channels at the PM (bottom) side, SERCA pumps at the ER (top) side, and a diffusion flow to the cytosol (lateral area).

Since detailed knowledge of the ER-PM junction is lacking accurate spatial modeling of the junctional calcium distribution requires certain assumptions. In general, the ER-PM junction is defined as the space between the PM and the ER. Following calcium depletion from the ER via RyR and IP3R channels, ER resident STIM1 proteins undergo conformational change, oligomerize, and translocate to ER-PM junctions, where they cluster to bind and activate the PM-resident ORAI1 channels and form the CRAC channels (see Figure 3A). During this process the STIM1 proteins remain anchored in the ER and cannot fully leave it, which implies that the thickness of the junction is partially limited by the length of the cytosolic STIM1, estimates for which vary between 10 nm to 20 nm (Islam, 2020). The ER does not have a smooth elliptical shape with a single large contact plane to the PM, but has a lamellar structure (Plattner and Hentschel, 2017) that comes into close contact with the PM in many small gaps. The small gaps are referred to as the ER-PM junctions in this work, whereas the sum of all junctions is called the ER-PM micro domain. Based on a detailed spatial approach of the junctional calcium [Ca^2+^]_Jun_ introduced by McIvor et al., 2018, it can be assumed that [Ca^2+^]_Jun_ near the PM is roughly at a constantly high level with a spike in the direct vicinity of the CRAC channels and then declines in a linear manner towards the edge of the junction, where it aligns with [Ca^2+^]_Cyt_. This is in line with the predictions from Hogan, 2015. Both approaches consider the junction as a single entity directly bounded by the cytosol or cytosolic calcium concentration. In this work, however, it is assumed that the junction is located within the microdomain and is surrounded by other junctions, which reduces the influence of the cytosol. As a consequence, we designed the ER-PM junction as a cylinder with a uniformly distributed calcium concentration inside and the ring surface acting as a diffusion layer with a linear diffusion profile towards Ca^2+^ cytosol, as shown in Figure 3B.

Inside the junction only the SERCA pump, the CRAC channels and the diffusion to the cytosol are considered to influence [Ca^2+^]_Jun_. All other possible ion channels or calcium binding proteins are not taken into account. With the current available knowledge (Hogan, 2015; McIvor et al., 2018), it is more feasible to directly model the calcium concentration of an individual junction rather than that of the microdomain, as this allows to circumvent the influence of other proteins and the irregularities of the ER surface. There are only estimations of how these junctions are actually shaped or sized. As there are no reports or measures for the junction dimensions of the A549 cell line, the measures are taken from other non-excitable cells instead (Hogan, 2015). The junctions are assumed to have a cylindrical shape, with a height between 10 nm and 20 nm and a diameter of 100 nm–300 nm. In other cell types, the micro domain is estimated to contain roughly 200 to 400 junctions (Hogan, 2015), which serves as a guiding value for this model.

#### 2.1.2 Mathematical description for the SOCE mechanism

The calcium concentrations of the three different compartments, cytosol ${\left[{Ca}^{2+}\right]}_{Cyt}$ , endoplasmic reticulum ${\left[{Ca}^{2+}\right]}_{ER}$ and the ER-PM junction ${\left[{Ca}^{2+}\right]}_{Ju}$ are described by a set of ODEs, which are designed based on continuity equations.

$$\frac{d{\left[{Ca}^{2+}\right]}_{Cyt}}{dt}=J_{IP3R}+J_{RYR}+J_{Leak}-J_{SERCA,Cyt}-J_{PMCA}+\frac{n_{Ju}}{V_{Cyt}}J_{Diff}-\frac{d{\left[{Ca}^{2+}\right]}_{B,Cyt}}{dt}$$ (1)

$$\frac{d{\left[{Ca}^{2+}\right]}_{ER}}{dt}=\frac{V_{Cyt}}{V_{ER}}\left(J_{SERCA,Cyt}-J_{IP3R}-J_{RYR}-J_{Leak}\right)+n_{Ju}\frac{V_{Ju}}{V_{ER}}J_{SERCA,Ju}+\frac{d{\left[{Ca}^{2+}\right]}_{B,ER}}{dt}$$ (2)

$$\frac{d{\left[{Ca}^{2+}\right]}_{Ju}}{dt}=J_{CRAC}-J_{SERCA,Ju}-\frac{1}{V_{Ju}}J_{Diff}$$ (3)

The terms J_x_ denote the calcium flow through the specified channel x, with calcium inflows having a positive sign and calcium outflows having a negative sign. The constants V_Cyt_, V_ER_ and V_Ju_ are the volumes of the cytosol, ER and junctions respectively, used to scale the ion flux between the domains and n_ju_ represents the number of assumed ER-PM junctions. The last terms at the end of Eqs 1, 2 describe the influence of the calcium buffers of the cytosol ${\left[{Ca}^{2+}\right]}_{B,Cyt}$ and the endoplasmic reticulum ${\left[{Ca}^{2+}\right]}_{B,ER}$ . The individual ion channel models that describe the gating behavior and open probability are summarized in detail in the Supplementary Material Part B.

Additionally, based on the concept provided by Liu et al. (Liu et al., 2010; Liu, 2012), a Ca^2+^ leak current J_leak_ between the ER and the cytosol, with constant flow rate R_leak_, driven by the concentration gradient between the ER and the cytosol, is included into the SOCE model:

$$J_{Leak}=R_{leak}\;\left({{\left[{Ca}^{2+}\right]}_{ER}-\left[{Ca}^{2+}\right]}_{Cyt}\right)$$ (4)

CRAC channels play a critical role in mediating store-operated calcium entry and are key channels in this process. What distinguishes CRAC channels from others is their property of being both calcium- and voltage-dependent. While many studies tend to focus only on either calcium or voltage dependence, here we consider both aspects in its mathematical description.

$$J_{CRAC}=n_{CRAC}\;g_{CRAC}{\;\rho }_{CRAC}\left({\left[{Ca}^{2+}\right]}_{ER}\right)*\left(V-E_{Ca}\right)*\frac{-1}{vcccj}$$ (5)

with $vcccj=\frac{1}{F\;z\;V_{Ju}}$ where the term (V-E_Ca_) describes the voltage dependence, V the membrane potential and E_Ca_ the Nernst potential for Ca^2+^ ions and vcccj a conversion constant that scales the current into an “ion flux”. The Nernst potential depends on the dynamic local [Ca^2+^] and is therefore updated for each time step:

$$E_{Ca}=\frac{RT}{zF}\ln\frac{{\left[{Ca}^{2+}\right]}_{Ext}}{{\left[{Ca}^{2+}\right]}_{Ju}}$$ (6)

where R is the universal gas constant, F the Faraday constant, z the elementary charge of Ca^2+^, T the temperature and [Ca^2+^]_Ext_ the extracellular calcium concentration.

It is known that CRAC channels are strongly inwardly rectifying under physiological conditions and therefore only allow calcium influx into the cell. To prevent a possible Ca^2+^ efflux under certain experimental conditions, the driving force is set to zero if an outward CRAC current is to be forced.

$$\left(V-V_{Ca}\right)=\left\{\begin{array}{c} \left(V-V_{Ca}\right)\;\forall \;\left(V-V_{Ca}\right)\;<0\\ \;0\;\forall \;\left(V-V_{Ca}\right)\geq 0 \end{array}\right.$$ (7)

If the potential difference is used as driving force in conjunction with the electrical conductance g_CRAC_, an electric current is obtained according to Eq. 5. This current is then converted into an “ion flux” - using the constant vcccj, which is defined as follows:

$$vcccj=\frac{1}{F\;z\;V_{Ju}}$$ (8)

where F is the Faraday constant, z the charge of calcium and V_Ju_ the volume of the target compartment, which in this case is the junction volume.

The formation of CRAC channels involves the interaction between the STIM1 protein in the endoplasmic reticulum and ORAI pore in the plasma membrane. According to Lui et al. (Liu et al., 2010; Liu, 2012), the activation process can be described in two steps. In the first step, when the endoplasmic concentration of calcium ions decreases, Ca^2+^ dissociates from STIM1, which leads to the activation of STIM1 (Liu, 2012).

$$n_{s}\left[{Ca}^{2+}\right]+\left[STIM1\right]\;\begin{array}{c} \overset{f_{s}}{\to }\\ \underset{b_{s}}{\leftarrow } \end{array}\;\left[{Ca}_{n_{s}}STIM1\right]$$ (9)

where [STIM1] describes the activated form and [Ca_ns_STIM1] the inactivated calcium bound molecule. To simplify the computation, the following relation is used (Liu et al., 2010):

$$\left[{Ca}_{n_{s}}STIM1\right]=\left[TotalSTIM1\right]-\left[STIM1\right]$$ (10)

Therefore, the reaction mechanism can be written as follows:

$$\frac{d\left[STIM1\right]}{dt}=-f_{s}\left[STIM1\right]{\left[{Ca}^{2+}\right]}_{ER}^{n_{s}}+b_{s}\left(\left[TotalSTIM1\right]-\left[STIM1\right]\right)$$ (11)

where f_s_ is the binding rate, b_s_ the dissociation rate and n_s_ a positive exponent (Liu et al., 2010). The activated STIM1 then spreads across the ER-PM junction, where it binds to the ORAI pore to form the CRAC channel, which is formulated as follows:

$$\left[STIM1\right]+\left[ORAI1\right]\;\begin{array}{c} \overset{f_{0}}{\to }\\ \underset{b_{0}}{\leftarrow } \end{array}\;\left[CRAC\right]$$ (12)

The differential equation for the CRAC channels can be derived similarly to the STIM1 equation.

$$\left[ORAI1\right]=\left[TotalORAI1\right]-\left[CRAC\right]$$

$$\frac{d\left[CRAC\right]}{dt}=f_{0}\left(\left[TotalORAI1\right]-\left[CRAC\right]\right)\left[STIM1\right]-b_{0}\left[CRAC\right]$$ (13)

Since, to our knowledge, no data are available for the total STIM1 and ORAI1 concentration, the initial values for [TotalSTIM1] and [TotalORAI1] are both set to 1. With this setting, the values for STIM1 and CRAC vary from 0 to 1 and can be interpreted as the relative amount of bound or unbound proteins, with the calculated CRAC value corresponding to the dimensionless relative amount of CRAC channels formed. Once the CRAC channels are formed, they are considered to be immediately open, so the CRAC variable indicates the relative number of open CRAC channels (Liu et al., 2010), i.e. the open probability for calculation of the CRAC current according to Eq. 14:

$${\;\rho }_{CRAC}\left({\left[{Ca}^{2+}\right]}_{ER}\right)=CRAC$$ (14)

#### 2.1.3 Model parameterization

The parameterization of the model is based on literature data and experimental data published elsewhere. In detail, the gating parameters for the CRAC, IP3R and RyR channel models were taken as stated in the original models (Sneyd and Dufour, 2002; Lee and Keener, 2008; Liu, 2012), with channel conductances or maximum flow rates varied to achieve a proper fit to the available experimental data. For the CRAC channel, the conductance was chosen to be the same as in the initial A549 *in silico* model (Zweifach and Lewis, 1993; Langthaler et al., 2021). The amount of CRAC channels per junction and the total number of junctions n_ju_ are based on literature data (Hogan, 2015; McIvor et al., 2018) and were adjusted to fit the experimental data in Padar et al., 2004; Hou et al., 2011. The parameters for the generic cytosolic buffer term and for the IP3 concentration were taken directly from Liu, 2012. For the calsequestrin buffer, parameters from Shannon et al., 2004 were used and the amount of calsequestrin was adjusted.

Conversion of electrical ion currents to particle flow rates requires the consideration of target volumes. A detailed report on the structure of A549 cells is provided by Jiang et al., 2010, according to which the cell diameter is 10.59–14.93 µm and the cell volume can be estimated to be 1670 μm^3^. The cellular volume can be divided into 28% nucleus and 72% cytoplasm, which can be roughly further subdivided into 4.6% mitochondria, 2.5% lamellar bodies, 1.39% lysosomes and the remaining cytoplasm. Since there are currently no estimates on the structure or size of the endoplasmic reticulum of the A549 cell, it is assumed that it occupies 5% of the cell volume. Typically, the junction dimensions are a height between 10 nm and 20 nm and a diameter of 100 nm–300 nm. In other cell types the micro domain is estimated to contain roughly 200 to 400 junctions, which serves as a guide value for this model, with 150 junctions selected to fit the experimental data. Assuming the junction to be a cylinder, the volume was calculated with a height of 20 nm and a diameter of 100 nm, as outlined in Table 2. The cytosolic volume is calculated by subtracting all listed sub volumes from the reported cell volume. The scaling factors were assessed based on the geometrical assumptions as described. The initial values for the differential equations were either assumed based on expected values or based on their original sources. The parameters and initial values used are summarized in Table 1 and Table 2.

**TABLE 1:** Initial values for calcium simulations.

Name	Value	Unit
Initial values		
[Ca]_Cyt,0_	0.064	µM
[Ca]_ER,0_	250	µM
[Ca]_Ju,0_	0.064	µM
[Ca]_b,0_	16	µM
IP3_0_	0.25	µM
[CSQ]_0_	7	µM
[STIM1]_0_	0	µM
[ORAI1]_0_	0	µM
RyR States	X_00_ = 0.998; X_10_ = 0; X_11_ = 0; X_10_ = 0.002	
IP3R States	R = 1; O = 0; A = 0; I1 = 0; I2 = 0; S = 0	

**TABLE 2:** Summary of model parameters.

Name	Value	Unit	Source	Name	Value	Unit	Source
Geometry				RyR Channel			
V_cell_	1670	µm^3	Jiang et al. (2010)	k_2_	0.045	µM1-2/s	Lee and Keener (2008)
V_Cyt_	1.1002∙10^–12^	L	calculated	k_-2_	60	1/s	
V_Jun_	7,854∙10^–20^	L	calculated	k_4_	0.47	µM^-1/s	
V_ER_	8.35∙10^–14^	L	calculated	k_-4_	5	1/s	
V_cell_/V_ER_	20	1	selected	k_6_	0.3	1/(µM s)	
jun_d	100	nm	selected	k_11_	0.0045	µM^-2/s	
jun_h	10	nm	selected	K_d1_	1000	µM	
n_Ju_	150	1	selected	K_d3_	10000	µM	
d_diff_	0.350	µm	selected	K_d8_	638.3	µM	
A_diff_	0.0031	µm^2^	calculated	k_7_ = k_4_			
				k_-6_ = k_-4_			
General Parameters				R_RyR_	200	µM/s	selected
Ca_ext_	1800	µM	selected	IP3R Channel			
F	96485.3329	As/mol		k_1_	0.64	1/(s µM)	Sneyd and Dufour (2002)
R	8.3144598	J/(mol K)		k_-1_	0.04	1/s	
T	273	K	Langthaler et al. (2021)	k_2_	37.4	1/(s µM)	
z	2	1		k_-2_	1.4	1/s	
V_m_	5	mV	Langthaler et al. (2021)	k_3_	0.11	1/(s µM)	
D_J_	220	µm^2/s	McIvor et al. (2018)	k_-3_	29.8	1/s	
				k_4_	4	1/(s µM)	
CRAC Channel				k_-4_	0.54	1/s	
n_s_	3		Liu (2012)	L_1_	0.12	µM	
f_s_	6.663*e−6	1/(µM3 s)		L_3_	0.025	µM	
b_s_	2.525	1/s		L_5_	54.7	µM	
f_o_	1.226	1/(µM s)		j_2_	1.7	1/s	
b_o_	0.06774	1/s		j_4_	1.7	1/(s µM)	
TotalSTIM1	1	µM		j_6_	4707	1/s	
TotalORAI1	1	µM		j_-2_	0.8	1/s	
g_CRAC_	24*e−15	S	Zweifach and Lewis (1993)	j_-4_	2.5	1/(s µM)	
n_CRAC_	5	1	Hogan, 2015; McIvor et al. (2018)	j_-6_	11.4	1/s	
Cytosolic Buffer				R_IP3R_	0.18	µM/s	selected
k_off_	500	1/s	Liu (2012)	IP3			
k_on_	100	1/(µM s)		R^ip^_ca_	2.8	µM/s	Liu (2012)
[Ca]_b,total_	660	µM		K^ca^_m_	1.1	µM	
Calsequestrin				R^ip^_d_	1	1/s	
[CSQ]_total_	14	µM	selected	[(IP3) ®]	0.25	µM	
k_D,CSQ_	638	µM	Shannon et al. (2004)	SERCA			
k_on,CSQ_	100	1/(µM s)		K_SERCA_	0.12	µM	selected
k_off,CSQ_	65000	1/s		I_SERCA_	0.025	1/µM	Liu (2012)
PMCA				R_SERCA_	100	µM/s	Liu (2012)
K_PMCA_	0.9	µM	selected	Leakage			
R_PMCA_	38	µM/s	Liu (2012)	R_leak_	0.002	µM/s	Liu (2012)

### 2.2 Available experimental data for verification of the SOCE model

To verify whether the SOCE model actually describes and reproduces the calcium dynamics in A549 cancer cells, the model is compared with calcium measurements reported in the literature. Two studies were found that conducted suitable experiments on calcium channels important for this model (Hou et al., 2011; Shin et al., 2018). The experiment by Hou et al., 2011 was chosen as a reference since it directly shows the function of the CRAC channels and indirectly the function of IP3R channels, RyR channels and PMCA pumps. (see Figure 5C in Hou et al., 2011). The measurement depicts the relative fluorescence, which allows for conversion to the calcium levels (Padar et al., 2004). However, as the fluorescence parameters are not clearly defined in this work, a direct relationship and thus a quantitative comparison with the calcium levels is not possible. In detail, the measurement starts in a calcium-free environment, with a constant fluorescence intensity, indicating equilibrium of the cytosolic calcium level. Approximately 1 minute later, 2 µM Thapsigargin (TG) is added in order to block the SERCA pumps. This causes a net calcium efflux from the ER via IP3R and RyR channels as well as the ER leak, that raises the cytosolic calcium. The outflow continues until the ER is depleted within 2 minutes. At this point all STIM1 proteins should be bound to ORAI1, resulting in the opening of all available CRAC channels. In a next step, the cells are perfused with 2 mM Ca^2+^, which leads to a rapid and large rise of the calcium level as calcium flows into the cytosol, predominantely via CRAC channels. The steep rise is followed by a plateau phase, indicating the maximum calcium inflow in the cytosol through the fully activated CRAC channels. As the SERCA channels remain blocked, the ER is further depleted of calcium and the CRAC channels stay open. The calcium influx is then terminated by the addition of 100 µM 2-APB, a store-operated calcium channel blocker, causing the calcium levels to decline to the resting level, probably mediated by calcium extrusion pumps such as PMCA. To reproduce this experiment, the developed model was set to an arbitrary starting point with an external calcium concentration of zero. Depending on the parameter setting, the calcium concentration lasts some seconds to minutes until a steady state is reached. Addition of drugs and external calcium is assumed to occur at time points 66s for the TG, 231s for the perfusion with calcium and 429s for the CRAC channel blockage (Hou et al., 2011). The modulation of the corresponding channels depending on the effect of the drugs administered is then simulated by adjusting the model parameters accordingly.

Another suitable experiment is reported by Shin et al., 2018, where compound K was used to trigger ER Ca^2+^ release in A549 cells, showing the effect of the drug on cytosolic calcium levels, the results of which are depicted in Figure 4A in Shin et al., 2018. In the first experiment, compound K is added to the cell in order to trigger ER Ca^2+^ release, which is indicated by a quick and permanent increase in the cytosolic calcium concentration. In a second experiment the cells are incubated before addition of the channel blocker dantrolene and BAPTA-AM, an intracellular Ca^2+^ chelator, to block RyR and IP3R channels, which reduces the apoptotic effect and prevents increase of cytosolic Ca^2+^. While it is not possible to simulate the effect of compound K, it is possible to compute a forced calcium release through RyR channels by increasing the channels' flow rate in order to reproduce the measured data by model simulations (Shin et al., 2018).

### 2.3 Implementation of the SOCE model and the combined A549-SOCE model

**FIGURE 4:**
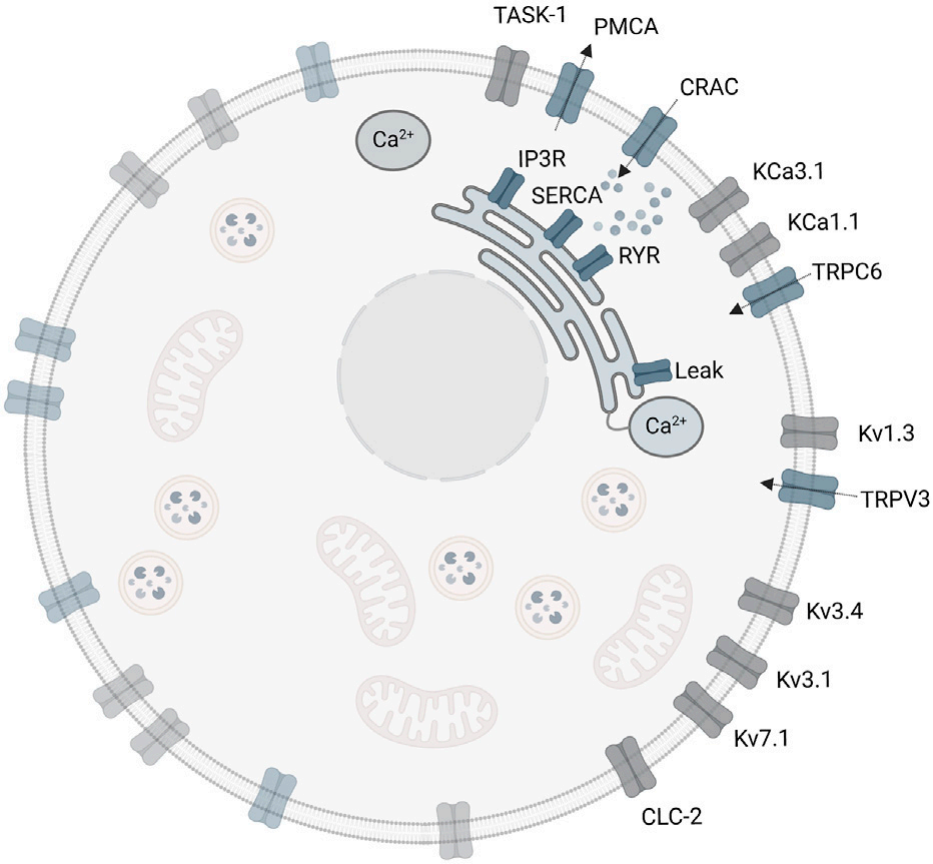
Extended A549-SOCE model by combining the initial plasma membrane channels with a detailed description of intracellular calcium channels and corresponding Ca^2+^ buffers in the endoplasmic reticulum and cytoplasm.

Implementation of the SOCE sub-model and the combined A549-SOCE model using MATLAB/Simulink (R2019b, MathWorks Inc.) is described in the Supplementary Material Part C. As in the initial A549 model, the equations are solved using the forward Euler method (ODE1 in Simulink), with a fixed step size of 5・10^−7^ s. The combined model is run on a workstation (2–4 × 256 GB RAM, CPU 2.20 GHz (Intel(R) Xeon(R) Fold 5220R), 48 cores, 96 logical processors), with a runtime of approximately 3–4 h for a 10 s simulation. The final A549-SOCE model approach is shown in Figure 4.

## 3 Results

### 3.1 SOCE model simulation and verification

**FIGURE 5:**
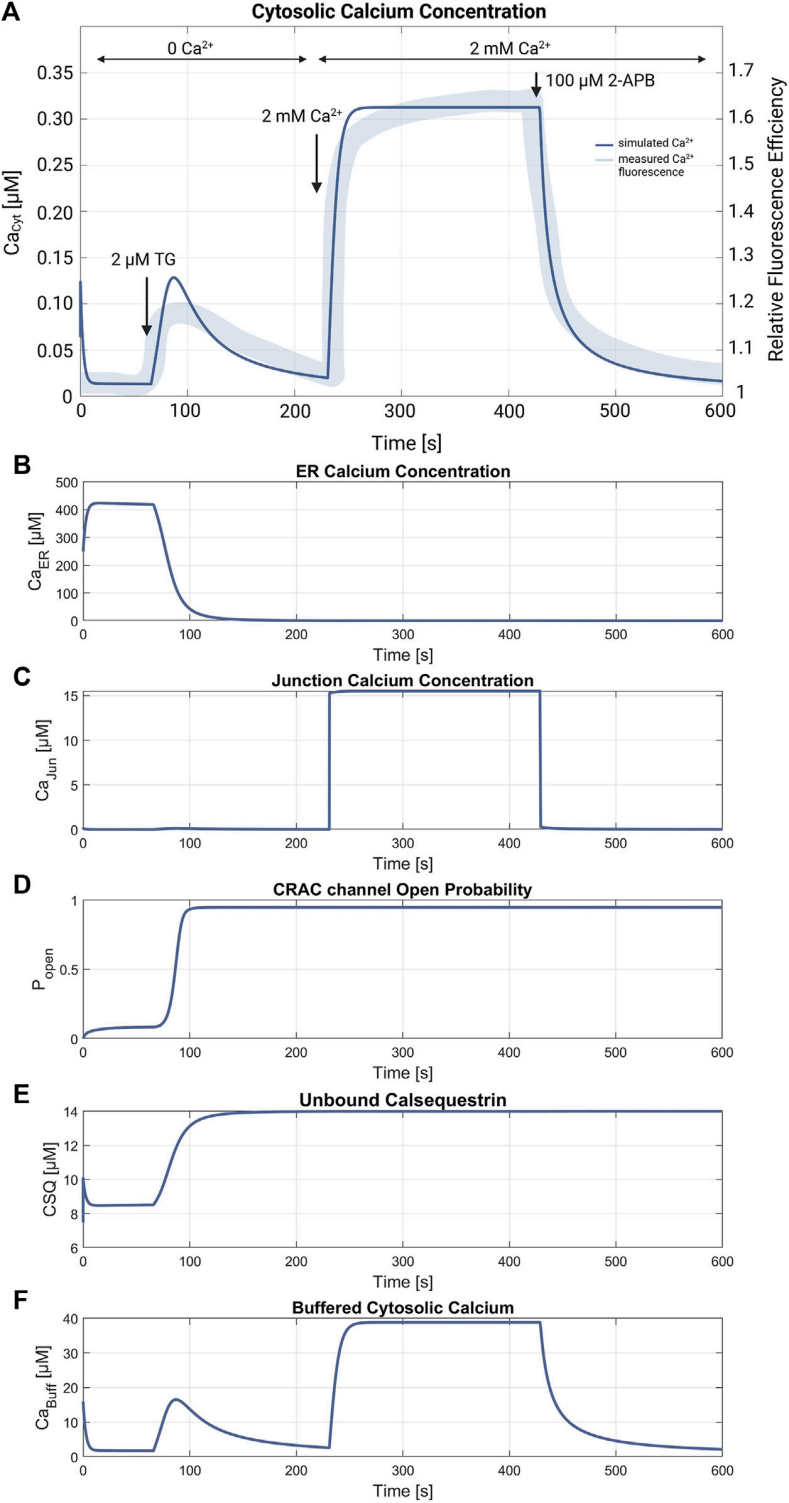
SOCE model verification. **(A)** Cytosolic calcium concentration simulation using the SOCE model (thin line in blue) superimposed with the measured calcium fluorescence from the experiment by Hou et al., 2011 (*cf.* Figure 5C in Hou et al., 2011) (thick line in blue). The arrows mark the addition of 2 µM Thapsigargin (TG) to block the SERCA pumps, start of perfusion of the cells with 2 mM Ca^2+^, and addition of 100 µM 2-APB, a store-operated calcium channel blocker. Simulation of the relevant SOCE parameters **(B)** [Ca^2+^]_ER_; **(C)** [Ca^2+^]_Jun_; **(D)** CRAC channel open probability; **(E)** Ca^2+^ unbound calsequestrin; and **(F)** buffered Ca^2+^ in the cytosol.

**FIGURE 6:**
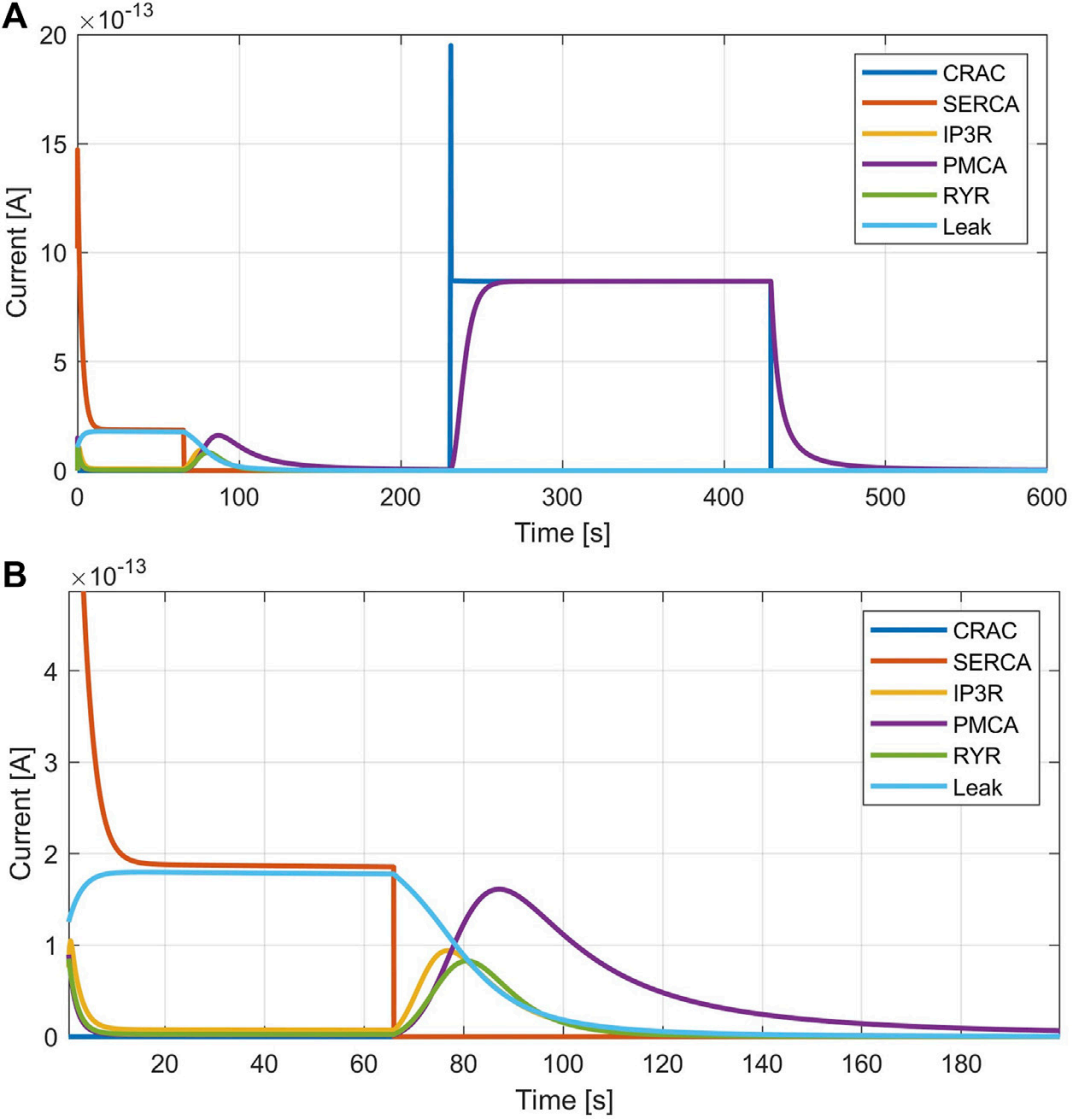
Calcium currents simulated with the SOCE model. **(A)** Currents throughout the entire simulation. **(B)** Zoom of SERCA pump inhibition.

Figures 5, 6 demonstrate the simulations for the settings of the SOCE concept (described in the Material and Methods section) based on the experiment by Hou et al., 2011 (*cf.* Figure 5C in Hou et al., 2011). Figure 5 shows a comparison of the cytosolic calcium concentration simulation *versus* the measured calcium fluorescence in the experiment. Note that the simulation yields an artificial steep calcium spike in the beginning, which reveals the transient response of the SOCE model setting to the calcium-free starting condition. Importantly, we emphasize that this transient response has no further impact on subsequent simulations. Once the initial phase is concluded, the simulation stabilizes, and the system reaches a physiologically relevant state. Despite all channels being active during this phase, the SERCA pumps are the most active, leading to a decrease of [Ca^2+^]_Cyt_ to a baseline of about 13 nM, while [Ca^2+^]_ER_ increases. The course of the buffered calcium inside the cytosol follows [Ca^2+^]_Cyt_ in shape, but with higher scaling. After 66 s, the flow rate of the SERCA pumps in the model is set to zero to simulate blockage by 2 μM TG application, resulting in a net outflow from the ER, and thus in a rapid decrease of [Ca^2+^]_ER_ and increase of [Ca^2+^]_Cyt_. During the ER efflux the IP3R and RyR channels open and increase calcium release. After reaching a temporal maximum of 128 nM, [Ca^2+^]_Cyt_ declines as the PMCA responds to the rising calcium levels and clears the cell of the remaining calcium. In this phase, the CRAC channels are activated by the decreasing ER calcium and reach an open probability of 95%. The addition of extracellular calcium causes an immediate transient current through the open CRAC channels, which settles at 0.87 fA after a short settling phase. The influx is followed by PMCA outflow, resulting in an almost constant calcium concentration of 334 nM. The calcium concentration of the junctions follows the course of [Ca^2+^]_Cyt_ with roughly the same values up to this point. The CRAC channel influx causes [Ca^2+^]_Jun_ to reach 15 µM during the plateau phase. The endoplasmic calcium and the CRAC channel open probability remain unchanged. The subsequent blocking of the CRAC channels is achieved by setting the number of CRAC channels per junction to 0.001, causing [Ca^2+^]_Cyt_ and [Ca^2+^]_Jun_ to decrease rapidly. Figures 5D–F summarize the simulations of the relevant SOCE parameters [Ca^2+^]_ER_, [Ca^2+^]_Jun_, CRAC channel open probability, buffered Ca^2+^ inside the cytosol, and Ca^2+^ unbound calsequestrin.

Figure 6 shows the currents of the ion channels in the simulated SOCE experiment. During the initial resting phase, ER efflux provoked by IP3R and RyR currents is almost zero and calcium leaves the ER mainly through the implemented leakage current. During the SERCA inhibition after 66 s, the leakage decreases, but both, the IP3R and RyR currents, increase in a bell-shaped fashion. While these channels are active, the ER is completely emptied of calcium, as illustrated by the decrease of [Ca^2+^]_ER_ and increase in unbound calsequestrin in Figures 5B,E. The calcium-free calsequestrin is capped at 14 μM, which is assumed to be the total amount of protein and means that no more calcium is buffered in the ER. The PMCA current starts at about the same time as the ER depletion, but is larger and decays much slower, similar to [Ca^2+^]_Cyt_. The addition of calcium leads to the opening of the CRAC and PMCA channels. Neglecting the spikes, i.e. the simulation artifacts caused by the direct supply of extracellular calcium, the CRAC and PMCA currents reach peak values of 0.9 pA. After CRAC blocking by 2-APB, only the PMCA current remains, which decreases rapidly. At the end of the simulation, all calcium compartments and buffers are almost empty and all calcium fluxes end.

### 3.2 Variation of model parameters

To evaluate how different parameter settings affect the model’s behavior, various simulations were carried out (see Supplementary Materia Part D **)**. The simulations in Supplementary Figures SD1, A–C are shown for the cytosolic calcium concentration, as this parameter can be measured experimentally. Each sub plot in the figure yields a variation of a single parameter, while one graph (in blue) serves as the reference simulation with the basic settings used for Figure 5. These simulations support the basic functionality of the proposed model description, as the mechanism of the CRAC channels and the junction model work properly.

### 3.3 Simulation of RyR channel mediated ER calcium release

**FIGURE 7:**
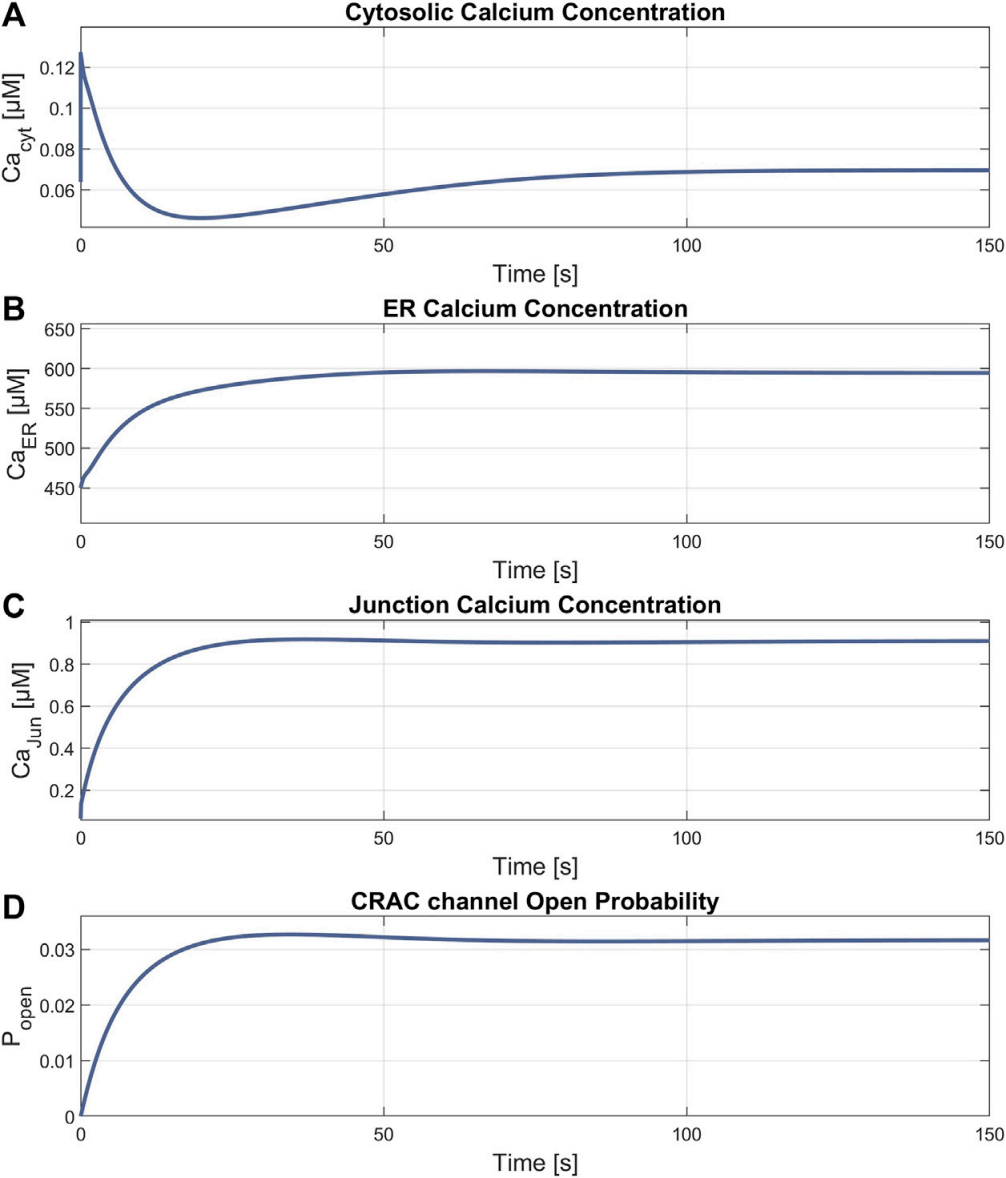
Results of the steady state simulation. Parameters with final values for **(A)** ${\left[{Ca}^{2+}\right]}_{Cyt}$ = 69 nM; **(B)** ${\left[{Ca}^{2+}\right]}_{ER}$ = 594 μM; **(C)** ${\left[{Ca}^{2+}\right]}_{Jun}$ = 0.9 µM and **(D)** ρ_CRAC_ = 0.03.

**FIGURE 8:**
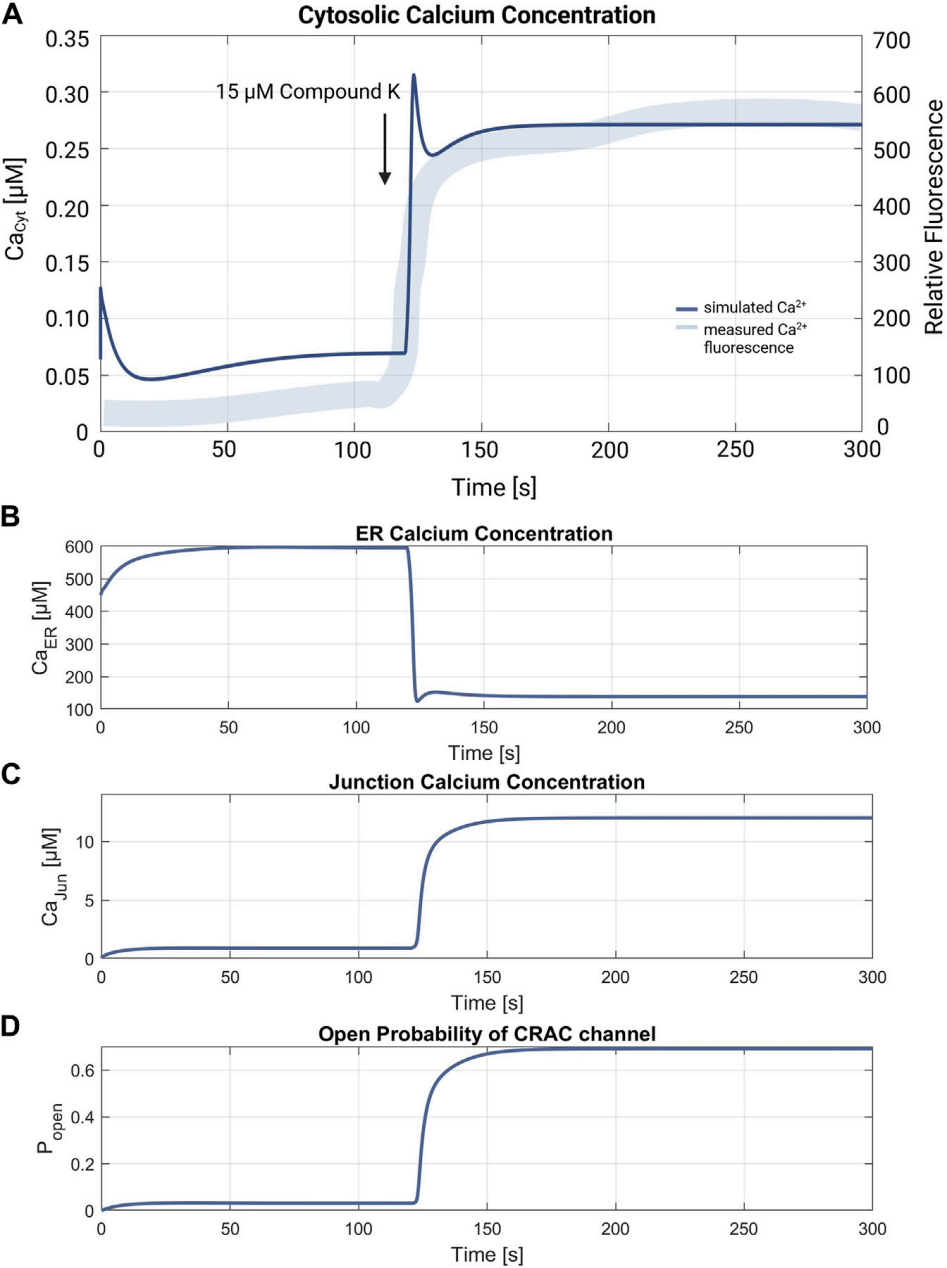
Verification of the compound K experiment by Shin et al., 2018. **(A)** Superimposition of the simulation of the cytosolic calcium concentration (thin line in blue) with the measured compound K concentration (thick line in blue). The arrow marks the addition of 15 µM compound K, leading to the RyR channel activation. The resulting cytosolic calcium level is ${\left[{Ca}^{2+}\right]}_{Cyt}$ = 272 nM. Simulations of **(B)** the ER calcium concentration, **(C)** the junction calcium concentration and **(D)** the open probability of the CRAC. Resulting calcium levels are ${\left[{Ca}^{2+}\right]}_{ER}$ = 139 µM and ${\left[{Ca}^{2+}\right]}_{Jun}$ = 12 µM. The final open probability of CRAC channels is ρ_CRAC_ = 0.69.

The simulation of the compound K experiment by Shin et al., 2018 as shown in Figure 8, starts in a steady state configuration. Hereto the SOCE model is run with an external calcium concentration of 1.8 mM for about 100 s, until steady state is reached**.** The resulting calcium concentrations with [Ca^2+^]_Cyt_ of 69 nM, [Ca^2+^]_ER_ at 660 μM, [Ca^2+^]_Jun_ at 0.9 µM and an open probability of the CRAC channels of 3% are shown in Figure 7. Note that the calcium concentrations depend on the parameter settings and thus result from the selected model parameterization. After reaching the baseline, the conductance of the RyR channel is increased by a factor of 10 at s 130, resulting in a steep increase of [Ca^2+^]_Cyt_ with a small overshoot that settles to a plateau after 20 s. The activation of RyR channels, rapidly depletes the ER of calcium and [Ca^2+^]_ER_ drops to 139 µM, as shown in Figure 8B. This, in turn, leads to an increased open probability of CRAC channels, which rises to 69% and lifts the junction calcium concentration [Ca^2+^]_Jun_ to 12 µM.

### 3.4 The combined A549-SOCE *in silico* model

To compare simulations of the combined A549-SOCE model with the original A549 simulations, the combined model was run in steady state configuration for the cell cycle phases G0, G1, S and G2-M. Therefore, the membrane potential V_m_ was simulated by numerically solving Eq. 15 for V_m_, starting at +5 mV until steady state was reached (t = 40 s, dt = 5.10^–7^):

$$C∙\frac{dV_{m}}{dt}=-I_{whole\_cell}$$ (15)

**FIGURE 9:**
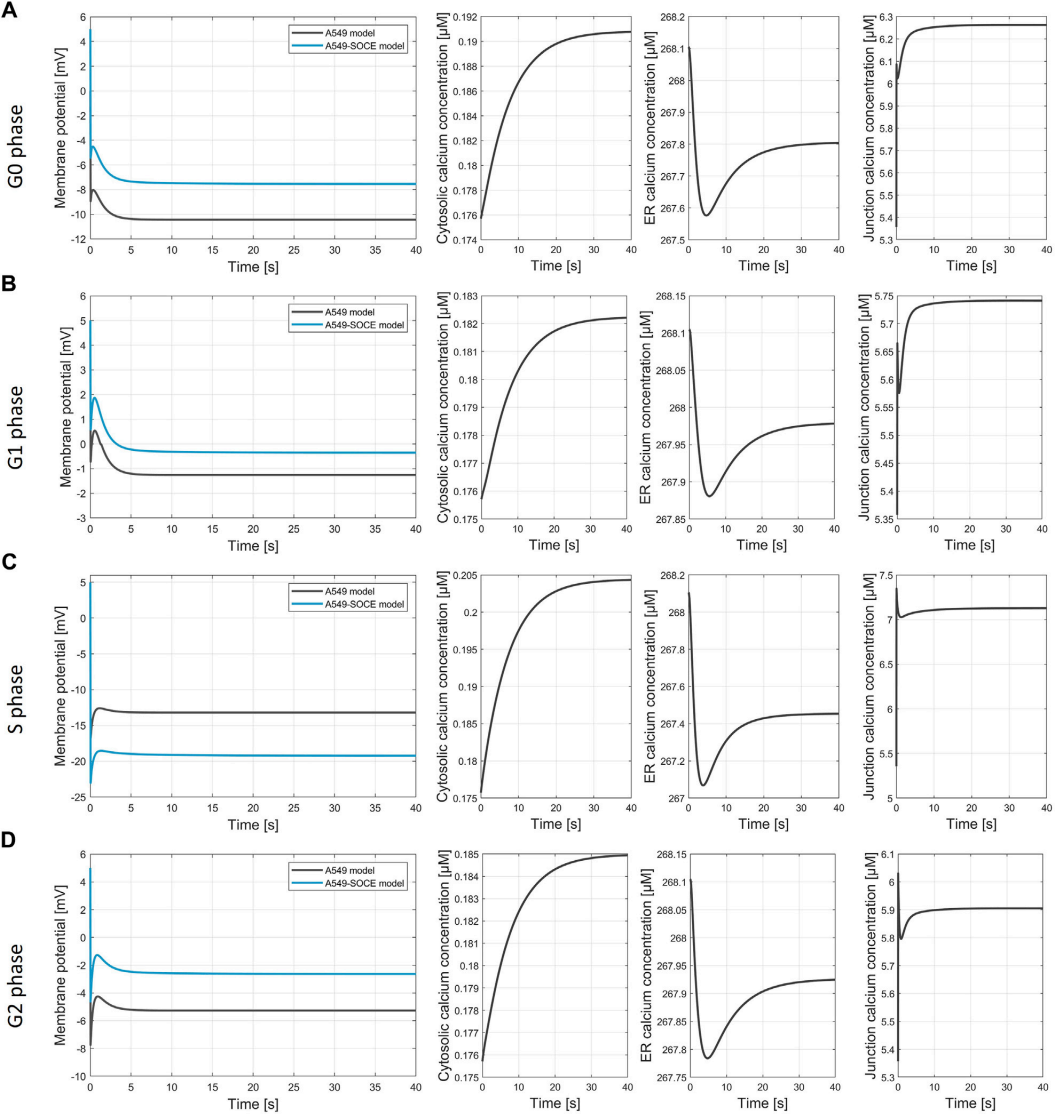
Simulation of the membrane potential during the cell cycle phases. **(A)** G0 phase, final values of the simulation V_m, A549_ = −10.4 mV; V_m.A549-SOCE_ = −7.5 mV; [Ca^2+^]_cyt_ = 0.191 µM; [Ca^2+^]_ER_ = 267.8 µM; [Ca^2+^]_jun_ = 6.263 µM; **(B)** G1 phase, final values of the simulation V_m, A549_ = −1.21 mV; V_m.A549-SOCE_ = −0.25 mV; [Ca^2+^]_cyt_ = 0.182 µM; [Ca^2+^]_ER_ = 267.98 µM; [Ca^2+^]_jun_ = 5.74 µM; **(C)** S phase, final values of the simulation V_m, A549_ = −13.2 mV; V_m.A549-SOCE_ = −19.1 mV; [Ca^2+^]_cyt_ = 0.204 µM; [Ca^2+^]_ER_ = 267.45 µM; [Ca^2+^]_jun_ = 7.13 µM; **(D)** G2 phase, final values of the simulation V_m, A549_ = −2.6 mV; V_m.A549-SOCE_ = −5.3 mV; [Ca^2+^]_cyt_ = 0.185 µM; [Ca^2+^]_ER_ = 267.92 µM; [Ca^2+^]_jun_ = 5.91 µM.

Figures 9A–D show the calculated membrane potentials of the combined A549-SOCE model and of the original A549 *in silico* model for the cell cycle phases G0, G1, S and G2-M with the plasma membrane ion channel composition as denoted in the original A549 model version (Langthaler et al., 2021) for comparison, without taking into account potential alterations in ion channel expression within the ER or Ca^2+^ depletion from the ER during distinct phases of the cell cycle. Since the open probability of the CRAC channels is lower with the new Ca^2+^ formalism, the number of CRAC channels was set to 1500 instead of 200 in the original A549 model in order to achieve comparable CRAC currents. In addition, [Ca^2+^]_Cyt_ and [Ca^2+^]_Jun_ are also shown to examine the state of the SOCE model.

**FIGURE 10:**
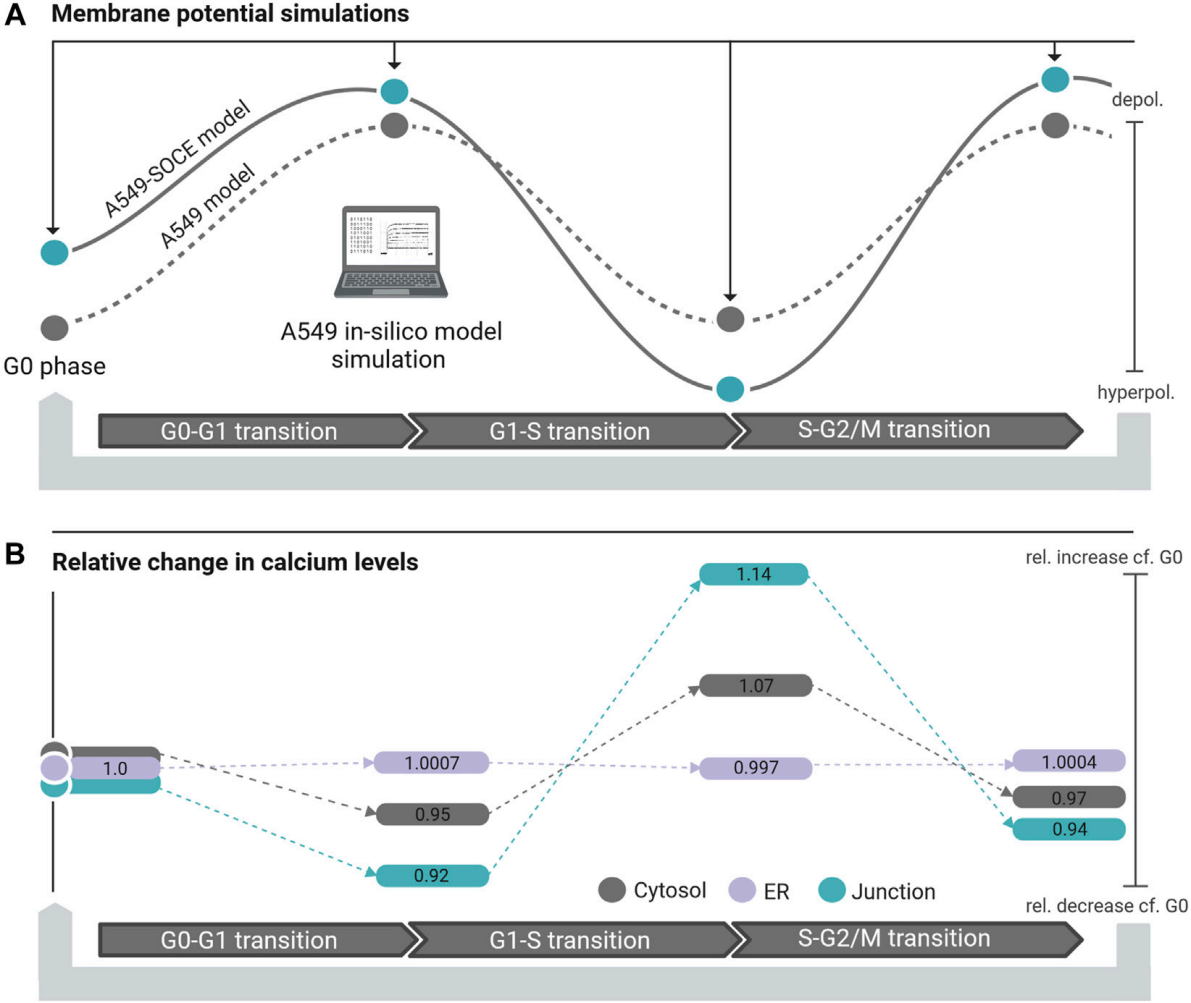
**(A)** Membrane potential simulation during cell cycle progression of the initial A549 model (dashed black line, grey dots simulated steady state potentials) and the A549-SOCE model (black line, blue dots simulated steady state potentials) showing the influence of calcium dynamics on the membrane potential in different phases of the cell cycle. **(B)** Relative change of the calcium concentration of the cytosol (grey bars), ER (purple bars) and junction (blue bars) in the different cell cycle phases normalized to the calcium concentrations in the resting G0 phase.

The membrane potentials as well as the response times of the combined model are roughly the same as those of the initial A549 model, as can be seen from the comparison of the membrane potential curves. For instance, the membrane potentials in G0 phase differ by less than 2 mV (V_m.original_ = −10.4 mV vs. V_m.calcium_ = −8.6 mV). A larger difference is seen in the S phase with V_S.diff_ = 5.9 mV (*cf.* Figure 9C). The combined model thus well reflects the oscillating characteristics of the membrane potential V_m_ during cell cycle progression (G0 phase: hyperpolarization, G1 phase: depolarization, S phase hyperpolarization, and G2-M phase depolarization)). Figure 10 outlines the rhythmic membrane potential change during cell cycle progression simulated with the initial and combined A549-SOCE model and relative changes of the calcium levels to the resting G0 phase. During the cell cycle phases, the cytosolic calcium levels fluctuate slightly between 0.182 and 0.204 µM and [Ca^2+^]_ER_ between 267.45 and 267.98 µM with the set parameters. The calcium levels of the junction are within 5.74 µM and 7.13 µM, which is higher than previously estimated by the initial A549 model. These high calcium levels result from increasing the maximum flow rate of the IP3R channels to 1.8 μM/s and the number of ER-PM junctions to 300, instead of the previously defined model parameters, to achieve currents in the expected sub nA range, as in the initial A549 model.

## 4 Discussion

### 4.1 The SOCE model

The SOCE model presented in this work allows the simulation and reproduction of the experimental data obtained by Hou et al., 2011 (*cf.* Figure 5C in Hou et al., 2011). However, the simulated calcium concentrations, in particular the initial cytosolic calcium concentration of [Ca^2+^]_Cyt_ = 13 nM could not yet be verified, as no experimental data in the absence of external calcium are available. In Padar et al. (2004) the baseline [Ca^2+^]_Cyt_ value of A549 cells is reported as 65 nM, presumably at an external calcium concentration of 1.2 mM. The [Ca^2+^]_Cyt_ levels reveal a relatively large difference but are in the same magnitude range, which indicates a reasonable model parameterization. The simulated ER calcium release matches the shape of SERCA blockage in the experiment in rise time and duration, but seems to have a steeper calcium decline. Since experimental values are missing, only a vague comparison of the simulated calcium plateau during the store-operated calcium entry is possible. Additionally, the ER Ca^2+^ release was compared with results of similar experiments of Padar et al., 2004, showing that the simulated data is consistent in terms of shape and time. However, using the stated relation and parameters for assessing the calcium concentration from the fluorescence response, the peak value of [Ca^2+^]_Cyt_ during ER depletion can be estimated to be about 360 nM, which is about three times the simulated peak value of 128 nM (Hou et al., 2011). This could indicate a higher calcium storage capacity of the ER in the real cell than assumed in the model simulations. The addition of external calcium leads to the activation of the store-operated calcium entry. The resulting [Ca^2+^]_Cyt_ during the SOCE plateau is 334 nM and thus very close to the expected [Ca^2+^]_Cyt_ of 300 nM determined by Hou et al., 2011. The other calcium compartments and the Ca^2+^ buffers equally demonstrate the expected behavior, but in contrast to [Ca^2+^]_Cyt_ cannot be further verified due to a lack of experimental data. The free calcium concentration of the ER is estimated to be in the range between 100 μM and 1 mM (Bong and Monteith, 2018; Rossi and Taylor, 2020), which matches the simulated values of almost 400 µM. However, the ER is completely emptied of free and bound Ca^2+^ during the ER depletion phase, which is reasonable from the simulation point of view, but may not be the case in a real cell considering the complex ER Ca^2+^ storage mechanisms (Islam, 2020). For [Ca^2+^]_Jun_, estimates and simulations assume values of 1 μM–7 µM for the overall junction and up to 60 µM in the immediate vicinity of the plasma membrane (McIvor et al., 2018). Thus, the resulting [Ca^2+^]_Jun_ of 15 µM in the simulation is higher, but within the realm of possibility. By contrast, the values for the Ca^2+^ buffers cannot be substantiated as data on CSQ inside A549 cells is lacking and the cytosolic buffer is a simplified construct that only describes the combined response of several intracellular proteins and organelles. Overall, however, as could be shown, the model reliably simulates the effects of SERCA blockage by Thapsigargin and thus aligns well with known effects on calcium dynamics in cells from an electrophysiological point of view.

Steady state simulations, as shown in Figure 7, settle at [Ca^2+^]_Cyt_ of 69 nM under 1.8 mM extracellular calcium, which agrees well to the 64 nM at 1.2 mM extracellular calcium reported by Padar et al., 2004. With less extracellular Ca^2+^, the intracellular Ca^2+^ also becomes smaller, so it can be assumed that the model parameters used slightly underestimate [Ca^2+^]_Cyt_. Nevertheless, the values are within the desired range, as [Ca^2+^]_ER_ settles at a high level of 594 μM, which indicates full Ca^2+^ stores.

The basic functionality of the model is underlined by additional parameter modulation shown in Supplementary Figures SD1, A–C **(** Supplementary Material Part D **),** as all parameter variations lead to the expected changes in simulated calcium concentrations. For example, increased IP3R and RyR conductivities in the simulation depict a faster Ca^2+^ efflux with a higher calcium peak, which is reasonable as these channels facilitate the ER efflux and more channels should result in a faster ion flux. Provided that no additional gating factors interfere, selective overexpression of these channels in a real cell should have a comparable effect which can therefore be simulated by the model approach. The number of junctions, ER leakage and diffusion distance constitute descriptions of cellular properties that are reasonable in a modeling context, but may not be directly applicable to a single feature in real cells due to abstraction. A valuable observation from these simulations is the relationship between [Ca^2+^]_Cyt_ and [Ca^2+^]_Jun_, which can be seen by comparing Supplementary Figures SD1, A and Supplementary Figures SD1, B. The number of junctions and the PMCA rate variations have approximately the same effect on the junction and the cytosol. The diffusion distance, on the other hand, has a much greater effect on [Ca^2+^]_Jun_ than on [Ca^2+^]_Cyt_ and can therefore be used to adjust [Ca^2+^]_Jun_ independently of its environment. In addition, the ER simulation results are an important output of the model. Starting with the IP3R channels, it appears that they do not change the resting state of [Ca^2+^]_ER_, but do affect the rate of ER efflux. Thus, the IP3R channel differs from RyR, which affects both the resting state [Ca^2+^]_ER_ and the rate of ER depletion. The leakage term has an unexpectedly large influence on the overall behavior, with too large leakage removing the Ca^2+^ from the ER and too small leakage significantly delaying the efflux. This shows that the ER leakage is an essential assumption for the model and is therefore probably also present in real cells. The increase of [Ca^2+^]_ER_ and the slower ER depletion with increasing amount of CSQ also represents a plausible simulation (see Supplementary Figures SD1, C) as CSQ represents a Ca^2+^ storage protein. The results of this variation suggests that the initial estimate of 14 µM CSQ was probably underestimated, as its influence on the model at concentrations below 140 µM appears to be small compared with other variables such as the cytosolic buffer capacity. A higher cytosolic buffer capacity, in turn, increases [Ca^2+^]_ER_, which could be explained by the higher total Ca^2+^ available in the cell. Finally, since the SERCA pumps are blocked at the beginning of the experiment, the effect of SERCA variation can only be investigated in the initial resting phase. Here, an increased pump rate leads to an increase in [Ca^2+^]_ER_, which also corresponds to the initial expectation.

Simulation of the experiment by Shin et al., closely matches the measured fluorescence response in time and shape, time and shape, thus reproducing the expected Ca^2+^ signaling chain for the CRAC channels. According to the experimental work, the simulation causes a permanent increase of [Ca^2+^]_Cyt_ due to the opening of RyR channels, as shown in Figure 8. Controlled Ca^2+^ depletion of the ER leads to the opening of CRAC channels and an increase of [Ca^2+^]_Jun_, which subsequently influences the corresponding ion channels until a steady state is reached. While the calcium overshoot at the beginning, which is caused by the fast current through the RyR channels, could indicate a too pronounced opening rate in the model, all other simulation components behave as expected.

Considering that the presented model was not fitted to quality-assured experimental data, the simulated absolute calcium concentrations of the SOCE experiment are within physiologically reasonable ranges. Since the shape and scale of the simulation results can be widely varied via its parameters, as shown in the simulations, it is to be expected that the model can be fitted to suitable experimental data and thus a better agreement with the experiments can be achieved. The model obtains a reliable simulation of the [Ca^2+^] course in the junction and it also reproduces the forced ER Ca^2+^ release through RyR channels, providing a realistic estimate for the Ca^2+^ environment in the resting state of the cell. Overall, it can be concluded that at this stage the model can simulate the necessary calcium handling pathways that influence Ca^2+^ distribution of the ER-PM junction. The actual Ca^2+^ levels within the junction are consistent with estimates reported in the literature and can be easily adapted to the needs of the simulation. Therefore, the model can be used to make a reliable estimate of the Ca^2+^ concentration within the ER-PM junction.

### 4.2 The combined A549—SOCE model

The initial A549 model was designed assuming fixed values of [Ca^2+^]_Cyt_ = 64 nM and [Ca^2+^]_Jun_ = 4.68 µM. The cytosolic Ca^2+^ is important for the driving force of Ca^2+^ permeable channels, and Ca^2+^ within the junction is assumed to regulate the opening of KCa channels. In the combined A549-SOCE model these two Ca^2+^ parameters are dynamically calculated. Integration of the of the SOCE model into the A549 model required further adjustments in order to fulfil the initial assumptions, which are described below.

Simulation of the SOCE model resulted in a [Ca^2+^]_Cyt_ of 69 nM in the resting state and a much lower [Ca^2+^]_Jun_ of 0.9 µM. This makes sense from the Ca^2+^ perspective, as a low [Ca^2+^]_Cyt_ with non-zero external calcium suggests full Ca^2+^ stores and therefore low CRAC activation and [Ca^2+^]_Jun_. However, the [Ca^2+^]_Jun_ proved to be too low to fully open the KCa channels, as assumed in the initial model, which requires [Ca^2+^]_Jun_ values above 5 µM. As a result, the current through the KCa channels was strongly reduced and the membrane potential depolarized considerably during first simulation trials. Hence, to ensure proper activation of KCa channels, the total calcium levels were increased by enhancing the maximum flow rate of IP3R channels. This has the dual effect of releasing more Ca^2+^ from the ER into the cytosol and increasing the influx of CRAC channels. As a second measure, the diffusion distance was adjusted to 350 nm for all simulations, increasing [Ca^2+^]_Jun_ independently of [Ca^2+^]_Cyt_. The resulting calcium levels of [Ca^2+^]_Jun_ and [Ca^2+^]_Cyt_ allowed a better simulation of KCa channel gating, according to original model assumptions.

Figures 9A–D demonstrate the simulation of the membrane potential of the extendend model. By integrating the SOCE model, the membrane potentials V_m_ during the G0 phase differ by 2.9 mV and those during the G1 phase by 0.96 mV from the original A549 model. The ion currents of the displayed channels also have comparable values to the original simulations (data not shown), so that no significant change in channel functions can be observed. Only the CRAC current is slightly below the original range, which results from the lower opening probability of the CRAC channels with the new Ca^2+^ formalism, despite the increased channel number from 200 to 1500. The Ca^2+^ levels both in the junction and in the cytosol are significantly higher in both cell cycle phases, but are within the normal range. The Ca^2+^ transients take about 20 s to reach a steady state, which is slower than the voltage dynamics and expected compared with the general Ca^2+^ and SOCE dynamics in cancer cells.

Similar observations to the results of the G0 and G1 phases can also be made for the S phase and G2/M phase, which is shown in Figures 9C,D. The deviation in V_m_ for S phase of 5.9 mV is larger and thus slightly greater than expected, but could certainly be improved by using advanced computational optimization methods.

In summary, the simulation confirms that the SOCE model is compatible with the initial A549 ion current model and leads to an appropriate response of the calcium dependent ion channels to simulated Ca^2+^ levels. In contrast to the variation of V_m_, the increasing cytosolic calcium concentration and the dynamic adaptation of the Ca^2+^ Nernst potential for the cytosol does not lead to a significant change in the driving force.

The new description of the CRAC channel provides a more profound model of CRAC gating, but its direct contribution to the whole-cell current is still small compared with the other Ca^2+^ channels. The important role of the CRAC channels for the model is regulation of the KCa channels. The initial model was designed with a high junctional calcium concentration, and thus, with almost fully open KCa3.1 channels, which was necessary to fit the experimental data. Although the high Ca^2+^ levels required for the simulation are within an acceptable range for cells, permanent activation of the CRAC channels is required to achieve the necessary calcium concentration for the KCa channels. However, the SOCE mechanism is only a temporary process limited to the time required to replenish the ER with Ca^2+^, and typical [Ca^2+^] levels in cells are much lower (Padar et al., 2004). In the case of lowering KCa3.1 activity, for example by reducing [Ca^2+^]_Jun_ due to the reduction of the diffusion barrierer, would lead to a depolarization of V_m_ above +10 mV. The problem could be explained by an insufficient KCa3.1 model that demonstrates a too low calcium dependence for the cell type. Another reason could be the data fitting of the initial model, as all cell cycle phases were fitted with the same [Ca^2+^] concentration. Similarly, the original assumption that the KCa channels are in close proximity to the CRAC channels may be incorrect. The KCa channels could instead be located near other calcium entry channels such as the TRP channels, which have a much higher conductance and could therefore generate higher local concentrations in their vicinity independent of the time limited SOCE mechanism. Nevertheless, more detailed data on the Ca^2+^ homeostasis are needed to allow adequate modeling of the actual KCa gating.

### 4.3 Model design and limitations

The entire SOCE concept of the developed model is based on several other model approaches. The regulatory mechanisms of the CRAC, SERCA, PMCA and IP3R channels are well known and have already been implemented with varying degrees of abstraction (Kowalewski et al., 2006; Means et al., 2006; Liu, 2012; Schmeitz et al., 2013; Eichinger et al., 2018). The novelty of this model lies in the unique combination of existing concepts and a higher level of detail. The most significant feature is the introduction of the ER-PM junction as a separate calcium domain, which has not been reported before in a similar form. Another sophisticated part is the addition of a kinetic state model for both the IP3R and RyR channels. While the IP3R channel has already been implemented as a kinetic state model (Means et al., 2006), this is typically an exception, and the RyR channel is generally neglected in SOCE models. Another notable sub-step is the Ca^2+^ and voltage dependency of the CRAC current. Although Ca^2+^ and voltage dependent CRAC current formalisms have been reported (Schmeitz et al., 2013; Eichinger et al., 2018), to the best of our knowledge, no model with the same level of detail was found in the literature reviewed.

An important geometric design decision is the diffusion barrier. It is generally assumed that the junction is directly surrounded by the cytosol on its side (Hogan, 2015; McIvor et al., 2018). However, the ER-PM junction is located in a microdomain with over 100 junctions, which means that the junctions that are not on the edge of the microdomain are not in direct contact with the cytosol. This would greatly enlarge the distance for the Ca^2+^ diffusion from the CRAC channels to the cytosol. If it is assumed that there is at least one junction with a diameter of 100 nm between the observed junction and the cytosol, the junctions are a few nm apart and the steady [Ca^2+^] decrease within the junction towards the edges is taken into account, then the diffusion distance for the junction would be around 120 nm–180 nm. This would mean that junctions in the center of the microdomain could have much larger diffusion distances and are almost isolated from the cytosol. With this assumption, a variable diffusion distance can be justified in the model, which can be set to determine [Ca^2+^]_Jun_. The diffusion area, i.e., the lateral surface of the cylindrical junction, also influences the diffusion, but it depends on the size of the junction and therefore cannot be varied as easily. The other variables of the basic diffusion relationship are the dynamic calcium domains and the fixed diffusion constant, making the diffusion distance the parameter of choice for adjusting the ion flux between the junction and the cytosol.

The main Ca^2+^ influx into the junction occurs via the CRAC channels. The number of CRAC channels per junction could be varied to modulate [Ca^2+^]_Jun_, but five channels is a number reported in the literature (McIvor et al., 2018) and was therefore retained for modeling. The gating formalism of the CRAC channels under the assumed simplification that the total amount of STIM1 and ORAI1 is simply 1 (µM) works as intended. The electrical driving force of the CRAC channels is regulated by the Ca^2+^ Nernst potential between the extracellular space and the junction. Simulation experiments during the development yielded much better results for a junction-based Nernst potential than for the [Ca^2+^]_Cyt_ -based one, as the rapid Ca^2+^ increase at the junction reduces the driving force and regulates the ion influx.

The chosen modeling approach for the SERCA is rather simple compared to other junction components, but it is sufficient to balance the calcium concentrations of the junction and cytosol and to fill the ER. No advanced kinetic state model has been found for the SERCA pump, and even the more detailed alternatives focus more on additional aspects such as the leakage (Means et al., 2006) or ATP dependency (Diederichs, 2008) rather than on the actual gating itself. The transport or reaction rate of the published SERCA models is typically only controlled by a single dissociation constant. The dissociation constant in this model does not represent real protein affinities, but was determined empirically and set to a value that yielded good results. Deviations from the real SERCA can be explained by the fact that there are different isoforms of SERCA pumps present in the cells (EMBL-EBI, 2023) and the formalism used represents an approximation of the combined behavior of different pumps with different Ca^2+^ affinities. Almost exactly the same observations and restrictions are made for the PMCA pump, with the difference that it only affects the cytosol. These pumps have the same basic functionality, are modeled by the same basic concept and give similarly satisfying results.

In contrast to SERCA and PMCA pumps, IP3R channels have already been described by various kinetic state models (De Young and Keizer, 1992; Sneyd and Dufour, 2002; Means et al., 2006). The modeling approach used provides expected results that fit well with the supposed SOCE mechanism. One limitation in modeling this channel is its dependence on the second messenger IP3. The highly simplified mathematical description of the IP3 dynamics used in this work does not reflect the biological processes that lead, for example, to an instant activation of the IP3R channel, but does allow a basic simulation of IP3R activation. Although such functionalities are beyond the scope of this first modeling approach, it must be considered a limitation. Similar model limitations also apply to the RyR channels, which are usually neglected in computational approaches. Although RyR channels play a minor role in ER Ca^2+^ release compared with IP3R, they are functionally expressed in A549 cells and can cause a significant ER Ca^2+^ efflux independently of IP3R (Shin et al., 2018). The addition of RyR channels therefore expands the possible simulation scenarios and represents the SOCE mechanism in more detail. These advantages justify the comparatively minor disadvantage of the additional computational effort in this stage.

The gating of the RyR model requires a description of the Ca^2+^ buffer CSQ, which leads to a further structural limitation of the model due to the introduction of buffer equations. The formalism for CSQ is appropriate and sufficient for RyR channel gating, but does not represent the buffered Ca^2+^ in the ER. Based on the results discussed it can be assumed that the initial amount of CSQ is probably too low and thus insufficient Ca^2+^ binds. It is also important to note that CSQ is not the major Ca^2+^ binding protein in the ER, and probably attributed to calreticulin (Means et al., 2006; Schmeitz et al., 2013), as a result of which the model does not simulate the buffered Ca^2+^ in the ER. In addition, the dimensioning of the entire ER is based on an assumption and the actual size of the ER could differ. Although the results for the Ca^2+^ inside the ER appear to be in the correct range and follow reasonable trends, these have to be interpreted with caution as they may deviate strongly from the actual Ca^2+^ environment of the real cell. The description of the buffer dynamics of the cytosol, on the other hand, represents a subsumption of the different Ca^2+^ binding proteins, which fulfills its purpose of shaping the Ca^2+^ dynamics to fit the reported experimental data but, like the CSQ description, does not accurately represent the amount of buffered Ca^2+^ of a real cell.

The TRP channels in the original A549 version are modeled as constant conductivities and therefore constantly open. The high conductivity causes large Ca^2+^ fluxes, which lead to a permanent overfilling of the SOCE model with Ca^2+^. For this reason, although the TRP channels significantly modulate the calcium concentration of the cells, they were excluded from the current SOCE model.

The main important limitation is the lack of experimental data for parameter fitting. Although the presented model can reproduce the SOCE mechanisms of the A549 cell line quite accurately in terms of time and shape of fluorescence intensity measurements, the calcium concentrations cannot be verified, which limits its predictive ability and reliability.

### 4.4 Outlook

To overcome existing limitations and improve accuracy, parameter fitting through computational optimization and a reparameterization of the A549-SOCE model is an essential next step, provided that data are available that allow quantitative simulations of the SOCE mechanism. For instance, recent studies have yielded valuable insights and successfully measured the free calcium concentration inside the ER lumen (Rossi and Taylor, 2020), which can serve as a reference for refining the ER description. In general, incorporating additional data concerning the Ca^2+^ homeostasis of A549 cells, such as resting [Ca^2+^] levels at different stages of the cell cycle, would certainly improve the model’s performance. Moreover, there’s a need to gain deeper knowledge of cell specific ion channel dynamics, such as TRP6, TRPV3, CRAC, and KCa channels. Developing new hidden Markov models or kinetic state models for these channels could significantly refine the SOCE model, potentially improving its accuracy in predicting parameters such as membrane potential (V_m_) and [Ca^2+^]_Jun_.

The presented SOCE model simulates the dynamics of the CRAC channel and the Ca^2+^ concentration specifically within the ER-PM junction. However, despite considering other Ca^2+^ domains, it is not intended as a general exhaustive representation of calcium dynamics encompassing all facets within the cell. Developing a comprehensive calcium model would necessitate a more profound description of the major Ca^2+^ buffer proteins in the ER and cytosol as implemented, e.g. in Means et al., 2006. The addition of other Ca^2+^ permeable channels or pumps, such as the Na^+^/Ca^2+^ exchanger would equally improve the accuracy of the model. A more sophisticated model should also include the mitochondria, which are the second largest intracellular Ca^2+^ store and, particularly important for the Ca^2+^ homeostasis. In addition, a detailed description of the IP3 cycle for IP3 mediated Ca^2+^ release in the ER or the possibility of simulating Ca^2+^ oscillations could be implemented. Some of these examples have already been described and implemented elsewhere and should be compatible with the developed SOCE model (Marhl et al., 2008; Liu, 2012; Schmeitz et al., 2013).

The presented model focusing only on electrophysiological processes, sets the stage for future explorations. The path toward enhancing the A549-SOCE model is multi-layered and involves optimizing the parameters, incorporating experimental data and validation, and, ultimately, extending the model to the downstream signaling pathways. This iterative process of refinement is fundamental in bridging the gap between theoretical modeling and biological reality, thereby laying the basis for novel, comprehensive tools in cancer research.

## 5 Conclusion

The developed model reliably describes the Ca^2+^ dynamics of the microdomain around the CRAC channels. It allows the simulation of store-operated calcium entry through the CRAC channels and shows the involvement of other Ca^2+^ channels and pumps during that process. The design of the ER-PM junction allows the user to customize the simulation to the needs of the application. The new SOCE model is compatible with the A549 *in silico* model and the new description of the Ca^2+^ concentration within the ER-PM junction can be used as a gating parameter for the KCa1.1 and KCa3.1 channels. The results of the combined A549-SOCE model indicate that the original assumption for the calcium concentration may have been too high, as evidenced by the results regarding the dynamics of the KCa3.1 channel. Despite these findings, it is now possible to make reasonable estimates for the Ca^2+^ environment of these ion channels, which may increase the accuracy of the whole-cell current model and its overall predictive capabilities. This work is partly limited by the fact that its parameters were not determined by optimizing experimental data, as these are only available to a limited extent. Future developments on the combined A549-SOCE *in silico* model should therefore include the parameterization of the SOCE model based on appropriate data and a general reparameterization of the ion current model with the new results presented in this work. Regardless of this, the model extension is an important step towards a profound and valid *in silico* model for oncological research and the development of novel therapeutic strategies supported by model simulations of digital cell twins.

## Data Availability

The original contributions presented in the study are included in the article/Supplementary Material, further inquiries can be directed to the corresponding author.
