# Supplementary material for: The bioelectric mechanisms of local calcium dynamics in cancer cell proliferation: an extension of the A549 in silico cell model
Source: Front Mol Biosci. 2024 May 6;11:1394398. doi: 10.3389/fmolb.2024.1394398 (PMC11102976; doi:10.3389/fmolb.2024.1394398)
Supplement: Supplementary file 1 [file DataSheet1.docx]

Supplementary Material

# Part A

## Calcium channels of the A549 cell line

The following is a summary of calcium ion channels in the human lung cancer cell line A549, the role and function of each ion channel and their effects on electrophysiological properties, importance for cell proliferation and tumor progression in A549 cells.

### CRAC channels

Induced overexpression of ORAI1/CRACM1 in A549 lung cancer inhibits SOCE and cell proliferation which leads to cell cycle arrest (Hou et. al., 2011). Inactivation of SOCE has been reported to inhibit cell proliferation and slow cell cycle progression in cervical and breast cancer cells (Chen et al., 2016). There are several studies suggesting a significant import of CRAC channels on cancer cells, which has consequently led to increased interest in them as potential targets for cancer therapy. Several CRAC channel inhibitors have been developed, for example SKF-96365, ML-9 or RP4010, showing promising anti-cancer effects in preclinical studies. However, due to a high toxicity of the drugs, no therapeutic application is currently available (Khan et al., 2020).

### Inositol 1,4,5-trisphosphate receptor (IP3R)

IP3R channels are located inside the ER membrane and function as Ca^2+^ release channels. Currently, three IP3R isoforms are known, IP3R Type 1, 2 and 3, all of which are expressed in the A549 cells (EMBL-EBI, 2023). These isoforms have a homology of 75% and differ in their sensitivity to various gating agents. The most important gating factors are IP3 and the surrounding [Ca^2+^] in the cytosol and the ER lumen (Islam, 2020). Thus, IP3R channels play a central role in the calcium signaling. For example, IP3R channels are essential for the Ca^2+^ supply of mitochondria, which require sustained Ca^2+^ release from the ER to replenish the internal [Ca^2+^] via the mitochondrial Ca^2+^ uniporter. This function alone provides a potential link between IP3R channels and apoptosis induction, as they can cause long lasting elevated Ca^2+^ levels in the cytosol and the mitochondria, which is considered toxic (Islam, 2020, Rezuchova et al., 2019). Thus, IP3R channels have been identified as key agents for the control of apoptotic processes and their involvement in these processes has been well documented for various cancer cells (Rezuchova et al., 2019). Depletion of ER stores in non-muscle cells is mainly facilitated via IP3R channels. Depending on the cell type, the depletion can occur either slowly over time or through cell activation as in human T-lymphocytes (Schmeitz et al., 2013). Extracellular signals such as growth factors or hormones can bind to tyrosine kinase receptors on the cell membrane and initiate a signaling cascade that activates calcium release via IP3R channels (Moccia, 2018). All these activations lead to lower ER Ca^2+^ levels, which subsequently activate the STIM proteins. It can be assumed that the SOCE process in non-excitable cells can be directly induced by store depletion via the IP3R channels. A detailed description of the IP3R channels is therefore essential for a sophisticated SOCE model.

### Ryanodine Receptor (RYR) channel

RYR channels are calcium release channels that are located within the ER membrane and, together with the IP3R channel, facilitate the Ca^2+^ efflux from the ER. There are three known mammalian RYR1-3 isoforms, of which RYR1 is expressed in A549 cells (EMBL-EBI, 2023, Lanner et al., 2010). RYR channels are crucial for the calcium signaling in muscle and neural cells and therefore very well described. In cardiac muscls, an action potential triggers voltage-gated calcium channels to increase the local Ca^2+^ concentration, which in turn activates the RYR channels that release even more calcium from the sarcoplasmic reticulum into the cytosol. This process, known as calcium induced calcium release, is a key mechanism that provides the cytosolic Ca^2+^ to interact with troponin C for muscle contraction. Besides, Ca^2+^ bind calmodulin to modulate smooth muscle contraction, gene transcription, metabolism, secretion, survival and proliferation in a wide range of cells (Berridge et al. 2000, Lanner et al., 2010).

Research on RYR channels is mainly limited to excitable cells, and only little information is available on their impact on cancer. It is generally assumed that IP3R channels are the dominant ER Ca^2+^ release channels in non-excitable cells and that RYR channels appear to play only a minor role. However, RYR channels are functionally expressed in A549 cells and it is shown that the manipulation of RYR channels impacts the Ca^2+^ distribution in A549 cells (Shin et al., 2018), which is why the channels were included in the model.

### Sarcoplasmic / endoplasmic reticulum Ca2+-ATPases (SERCA)

The SERCA pumps are located in the membrane of the ER and pump Ca^2+^ from the cytosol into the ER. SERCA pumps are ubiquitously expressed in most mammalian cells, but the expressed isoforms differ depending on the cell types. SERCA1 and SERCA2a are expressed in muscle cells, whereas SERCA2b and SERCA3 are found in smooth muscle or non-muscle cells (Lytton et al., 1992). The latter two isoforms are expressed in the A549 cells (EMBL-EBI, 2023). The pumps are assumed to be evenly distributed throughout the ER, and estimates for the RBL-2H3 cell line suggest that one cell contains approximately 1.6 million SERCA units. This large number is paired with the very low current of 0.005fA, which roughly corresponds to a transport rate of 16 Ca^2+^ per second (Means et al, 2006), or 36 Ca^2+^ per second for SERCA2b according to (Hogan, 2015). SERCA channels are essential for calcium homeostasis, as they keep [Ca^2+^]_Cyt_ low and fill the ER with Ca^2+^, creating the electrochemical gradient that drives the controlled Ca^2+^ release via the IP3R and RYR channels. It should be noted that SERCA pumps should not be considered as simple IP3R or RYR antagonist. There are experimental observations showing reduced calcium release in inhibited SERCA channels, suggesting that the pump is also involved in the Ca^2+^ release process or Ca^2+^ leakage (Islam, 2020). and has direct influence on the calcium distribution in the ER-PM junctions as they deplete the junction of calcium during SOCE. It can be assumed that the number of SERCA per junction is sufficient to accommodate the Ca^2+^ influx of the CRAC channels, which corresponds to approximately 700 pumps per junction (Hogan, 2015).

As the only Ca^2+^ transporter into the ER, SERCA channels are a central element of the cellular Ca^2+^ regulation and thus crucial for the regulation of cell survival and apoptosis. The involvement of irregular SERCA function in various diseases and cancer progression has been extensively reviewed in the literature (Chemaly et al., 2018). In A549 cells it has been experimentally documented in various reports (Padar et al., 2004, Hou et. al., 2011, Tatur et al. 2007), whereby the inhibition of SERCA pumps by the channel blocker thapsigargin leads to impaired actin cytoskeletal dynamics and thus apoptosis (Wang et al, 2014).

### Plasma Membrane Ca^2+^-ATPases (PMCA)

The PMCA pump regulated by ATP, pumps Ca^2+^ from the cytosol into the extracellular space. Together with the Na^+^/Ca^2+^ ATPase, which also transports Ca^2+^ out of the cell, it regulates the basal cytoplasmic calcium concentration and limits free calcium spikes (Roberts-Thomson et al., 2010). PMCA is encoded by the ATP2B1-4 genes and has four isoforms PMCA1-4, of which the isoforms PMCA1 and PMCA4 are expressed in the A549cells (EMBL-EBI, 2023). Due to alternate splicing of the genes, more than 20 variants of the protein are known. These different PMCA variants have different activation rates and enable a broad spectrum of regulatory interactions with other messengers, making PMCA an integral part of the calcium signaling (Islam, 2020). One regulatory factor is the calcium buffer molecule calmodulin, which lowers the Ca^2+^ affinity of PMCA from 10 µM to 0.2 µM, allowing the pump to function under physiological conditions. The PMCA hydrolyses one ATP to simultaneously transfer one Ca^2+^ ion out of the cell and two H^+^ ions into the cell. This makes the PMCA electrically neutral with respect to the membrane potential, which is important as it is located in the plasma membrane, whereby its current must be neglected when calculating the whole cell current (Thomas, 2009).

Compared to the other calcium channels discussed here, PMCA has received less attention in pathology and cancer research, but there are some studies reporting altered PMCA expression level in cancer cells that may be related to tumor progression. Deregulated PMCA1 and PMCA4 expression has been detected in transformed SV40 skin and lung fibroblasts as well as in oral-, colon- and breast cancer cells (Roberts-Thomson et al., 2010). One study suggests that the expression of PMCA2 in breast cancer cells could be an indicator for a better survival prognosis (Peters et al., 2016). Whether or not PMCA alone is an important contributor to the hallmarks of tumor progression is debatable, as there is little to no clear evidence, but it certainly influences the calcium homeostasis of the tumor cells.

### Transient receptor potential (TRP) channels

TRP channels are a large group of cation permeable, non-selective ion channels that activate in response to a variety of stimuli such as temperature, tension, pH or pheromones (Clapham, 2003). The channel family has an overall homology of only 20% and is divided into seven main subfamilies, including the TRPC (TRP Canonical) or TRPV (TRP Vanilloid) group (Owsianik et al., 2006). The two channels TRPC6 and TRPV3 are included in the A549 in-silico model.

TRPC6 are generally thought to be highly Ca^2+^ permeable with a Na^+^:Ca^2+^ conductivity ratio of 1:5. However, there is a report that the actual Ca^2+^ current is much lower with a Na^+^:Ca^2+^ ratio of only 25:1 under normal extracellular conditions (Estacion et al., 2006). TRPC6 channels are not activated by depletion of Ca^2+^ stores or by intracellular dialysis with IP3 (Estacion et al., 2006), and therefore not relevant in the context of SOCE. TRPC channels are generally not store-operated channels, but some of them have been shown to be associated with SOCE (Kozak and Putney, 2018). One well-described channel is TRPC1, which interacts with STIM proteins and can form a STIM1-ORAI1-TRPC1 ternary complex. TRPC1 channels can be assumed to influence the functionality of CRAC channels during SOCE, but no specific mechanisms have yet been confirmed (Lopez et al., 2020).

TRPV3 is a temperature sensitive ion channel with high temperature sensitivity at the physiological point at 37°C (Xu et al., 2002). The channel has been reported to be more permeable to Ca^2+^ than Na^+^ with a Ca^2+^:Na^+^ ratio ranging from 5:1 (Xu et al., 2002) to 11:1 (Owsianik et al., 2006). None of the reviewed articles mention a potential link between the TRPV3 channel and SOCE, so there may not be no involvement or none has yet been discovered.

# Part B

### Model of the Sarco/Endoplasmic Reticulum Calcium ATPase (SERCA)

The SERCA pump transports calcium ions against the concentration gradient from the cytosol to the ER. Its aim is to keep [Ca^2+^]_Cyt_ low and to fill the ER with Ca^2+^. The pump is normally driven by the local calcium concentration and ATP. There are ATP dependent models for the SERCA pump available (Diederichs, 2008). The influence of ATP is not considered here, as it is assumed to be present in sufficiently high concentrations at all times with which theflow of the SERCA pump can then be described by a Hill function (Liu, 2012, Kowalewski et al., 2006):

$J_{SERCA,Cyt}=\frac{R_{SERCA}\left[ {Ca}^{2+} \right]_{Cyt}}{K_{SERCA}+\left[ {Ca}^{2+} \right]_{Cyt}}*\frac{1}{1+ I_{SERCA} \left[ {Ca}^{2+} \right]_{ER}}$ (B1)

The first term on the right-hand side of the equation describes the activity of the pump, where R_SERCA_ is the maximal pump activity and K_SERCA_ the half saturation constant. The second term is a feedback controller that reduces the SERCA activity when [Ca^2+^]_ER_ becomes too high (Liu, 2012).

Two separate SERCA flows are implemented, one from the cytosol to the ER called J_SERCA,Cyt_ and one from the junction to the ER called J_SERCA,Jun_. For J_SERCA,Jun_ the calcium concentration at the junction is used as an independent variable in the Hill function. This is necessary because the local calcium concentrations in these two regions can be very different, which leads to different ion fluxes.

### Model of the Plasma Membrane Calcium ATPase (PMCA)

The PMCA pumps calcium from the cytosol into the extracellular space against the concentration gradient in order to keep the intracellular free calcium at a low level. Like the SERCA pump, the PMCA can also be modeled with a Hill equation according to (Liu, 2012, Kowalewski et al., 2006):

$J_{PMCA}=\frac{R_{PMCA}\left[ {Ca}^{2+} \right]_{Cyt}^{2}}{K_{PMCA}^{2}+\left[ {Ca}^{2+} \right]_{Cyt}^{2}}$ (B2)

Here, R_PMCA_ is the maximal pump activity and K_PMCA_ is the half saturation constant. In this case, no feedback term is required as it can be assumed that the extracellular space is independent of the cellular processes (Liu, 2012).

### Model of the ryanodine receptor (RYR) channel

The RYR channel allows the efflux of calcium from the ER, which is controlled by the calcium concentration gradient between the ER and the cytosol. This type of channel has been well studied for muscle cells for which corresponding models exists. Unfortunately, there is no RYR model for non-excitable cells, so a muscle cell model presented by Lee and Keener, 2008 is used. The gating of the RYR channel was implemented according to this reference, but the flow rate was adapted to the experiments. The calcium flux through the RYR channels is defined as:

$J_{RYR}=v_{1}\rho_{RYR}\left( \left[ {Ca}^{2+} \right]_{Cyt},[CSQ] \right)\left( {\left[ {Ca}^{2+} \right]_{ER}-\left[ {Ca}^{2+} \right]}_{Cyt} \right)$ (B3)

Here, v_1_ is the maximal channel permeability and P_RYR_ is the open probability for the RYR channels. P_RYR_ is calculated with a four-state kinetic model (Lee and Keener, 2008, **Figure B1**).


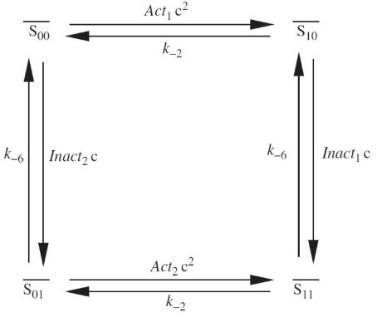


Supplementary Figure B1: Kinetic model of the RYR channel gating. The nodes Sxx represent the states Xxx. The transitions between the states depend on the activation, inactivation and transition constants (Lee and Keener, 2008).

The nodes S00, S01, S10 and S11 represent the states X00, X01, X10 and X11. The state variable X10 corresponds to the open state, which can be directly used as the open probability of the RYR channel (Lee and Keener, 2008).

$\rho_{RYR}=x_{10}$ (B4)

The states are described by a set of ODEs.

$\frac{dx_{00}}{dt}=\left( k_{-2}x_{10}-\left[ {Ca}^{2+} \right]_{Cyt}^{2}{Act}_{1}x_{00} \right)+\left( k_{-6}x_{01}-\left[ {Ca}^{2+} \right]_{Cyt}{Inact}_{2}x_{00} \right)$ (B5)

$\frac{dx_{10}}{dt}=\left( {\left[ {Ca}^{2+} \right]_{Cyt}^{2}Act}_{1}x_{00}-k_{-2}x_{10} \right)+\left( k_{-6}x_{11}-\left[ {Ca}^{2+} \right]_{Cyt}{Inact}_{1}x_{10} \right)$ (B6)

$\frac{dx_{11}}{dt}=\left( \left[ {Ca}^{2+} \right]_{Cyt}{Inact}_{1}x_{10}-k_{-6}x_{11} \right)+\left( \left[ {Ca}^{2+} \right]_{Cyt}^{2}{Act}_{2}x_{01}-k_{-2}x_{11} \right)$ (B7)

$\frac{dx_{01}}{dt}=\left( k_{-2}x_{11}-\left[ {Ca}^{2+} \right]_{Cyt}^{2}{Act}_{2}x_{10} \right)+\left( \left[ {Ca}^{2+} \right]_{Cyt}{Inact}_{2}x_{00}-k_{-6}x_{01} \right)$ (B8)

where Act_x_ and Inact_x_ are the activation and inactivation factors, and k_x_ the transition constants between the states. The transition constants k_x_ depend on the binding of calcium on the cytosolic side and of Ca^2+^ free calsequestrin on the ER side. The model considers two calcium binding sites, one for the channel activation, which binds two calcium ions, and a second one for inactivation, which binds one ion. The concentration of the calcium free calsequestrin [CSQ] acts as an inhibitor for the RYR channel, its binding therefore decreases the channel activity. The [CSQ] dependence is implemented via activation and inactivation factors. These factors are determined by [CSQ], the dissociation constants K^d^ and the rate constants k_x_ according to Lee and Keener, 2008.

| ${Act}_{1}=\frac{k_{2}K_{1}^{d}+k_{11}[CSQ]}{K_{1}^{d}+[CSQ]}$ | ${Act}_{2}=\frac{k_{2}K_{8}^{d}+k_{11}\left[ CSQ \right]}{K_{8}^{d}+\left[ CSQ \right]}$ |
| --- | --- |
| ${Inact}_{1}=\frac{k_{6}K_{3}^{d}+k_{7}\left[ CSQ \right]}{K_{3}^{d}+\left[ CSQ \right]}$ | ${Inact}_{2}=\frac{k_{6}K_{1}^{d}+k_{7}[CSQ]}{K_{1}^{d}+[CSQ]}$ |

### Model of the inositol (1, 4, 5) triphosphate receptor (IP3R) channel

The IP3R channel is modeled in a similar way to the RYR channel. It is a calcium release channel of the ER and its calcium flux is controlled by the calcium concentration gradient between ER and cytosol.

$J_{IP3R}=R_{IP3R}\rho_{IP3R}\left( \left[ {Ca}^{2+} \right]_{Cyt},[IP3] \right)\left( {\left[ {Ca}^{2+} \right]_{ER}-\left[ {Ca}^{2+} \right]}_{Cyt} \right)$ (B9)

R_IP3R_ is the maximum channel permeability and P_IP3R_ the open probability of the IP3R channels, which is determined by a model adopted from Sneyd and Dufour, 2002. It comprises five states that depend on the cytosolic calcium concentration and on the concentration of the second messenger IP3. The IP3 concentration is determined by a separate equation, which is explained in section “Model of the inositol 1,4,5-triphosphate (IP3) concentration”. The kinetic scheme of the model is shown in **Figure B2**.


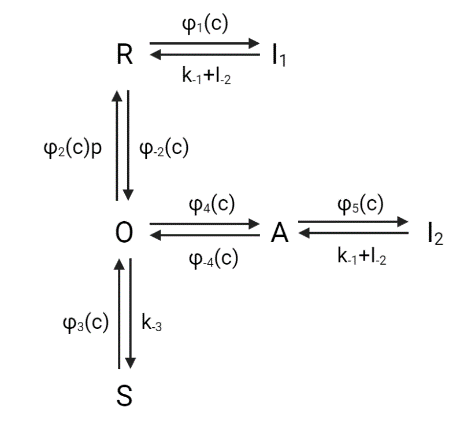


Supplementary Figure B2: Kinetic five-state model of the IP3R channel introduced by Sneyd and Dufour, 2002. Here $\boldsymbol{\phi}_{\mathbf{x}}$ denotes the transition rates between the states, c the cytosolic calcium and p the IP3 level.

It is assumed that the channel conducts Ca^2+^ in the open (O) and activated (A) states and is closed in the other states. It is further assumed that the IP3R channel consists of four independent sub units and that the open probability depends on their combined activity (Sneyd and Dufour, 2002).

$\rho_{IP3R}=\left( 0.1 O+0.9 A \right)^{4}$ (B10)

The nodes of the model represent the state of the same symbol and can be written as a series of ODEs. The transitions between the states depend on [IP3], the rate constants k_x_ and l_x_ and the [Ca^2+^]_Cyt_ dependent rates φ_x_.

$\frac{dR}{dt}=\phi_{-2}O-\phi_{2}\left[ IP3 \right]R+\left( k_{-1}+l_{-2} \right)I_{1}-\phi_{1}R$ (B11)

$\frac{dO}{dt}=\phi_{2}\left[ IP3 \right]R-\left( \phi_{-2}+\phi_{4}{+\phi}_{3} \right)O-\phi_{-4}A+k_{-3}S$ (B12)

$\frac{dA}{dt}=\phi_{4}O-\phi_{-4}A+\phi_{5}A+\left( k_{-1}+k_{-2} \right)I_{2}$ (B13)

$\frac{dI_{1}}{dt}=\phi_{1}R-\left( k_{-1}+l_{-2} \right)I_{1}$ (B14)

$\frac{dI_{2}}{dt}=\phi_{5}A-\left( k_{-1}+l_{-2} \right)I_{2}$ (B15)

$\frac{dS}{dt}=\phi_{3}O-k_{-3}S$ (B16)

The transition rates φ_x_ include the calcium dependencs of the model. They are calculated using [Ca^2+^]_Cyt_ and the rate constants k_x_ and l_x_, which are given by activity (Sneyd and Dufour, 2002):

| $\phi_{1}=\frac{\left( k_{1}L_{1}+l_{2} \right)\left[ {Ca}^{2+} \right]_{Cyt}}{L_{1}+\left[ {Ca}^{2+} \right]_{Cyt}\left( 1+\frac{L_{1}}{L_{3}} \right)}$ | $\phi_{2}=\frac{k_{2}L_{3}+l_{4}\left[ {Ca}^{2+} \right]_{Cyt}}{L_{3}+\left[ {Ca}^{2+} \right]_{Cyt}\left( 1+\frac{L_{3}}{L_{1}} \right)}$ | $\phi_{-2}=\frac{k_{-2}+l_{-4}\left[ {Ca}^{2+} \right]_{Cyt}}{1+\frac{\left[ {Ca}^{2+} \right]_{Cyt}}{L_{5}}}$ |
| --- | --- | --- |
| $\phi_{3}=\frac{k_{3}L_{5}}{L_{5}+\left[ {Ca}^{2+} \right]_{Cyt}}$ | $\phi_{4}=\frac{\left( k_{3}L_{5}+l_{6} \right)\left[ {Ca}^{2+} \right]_{Cyt}}{L_{5}+\left[ {Ca}^{2+} \right]_{Cyt}}$ | $\phi_{-4}=\frac{L_{1}\left( k_{-4}+l_{-6} \right)}{L_{1}+\left[ {Ca}^{2+} \right]_{Cyt}}$ |
| $\phi_{5}=\frac{\left( k_{1}L_{1}+l_{2} \right)\left[ {Ca}^{2+} \right]_{Cyt}}{L_{1}+\left[ {Ca}^{2+} \right]_{Cyt}}$ |  |  |

### ER leak current

Based on the concept of Liu et al. (Liu, 2012, Liu et al., 2010), a Ca^2+^ leak current between the ER and the cytosol is included in the SOCE model of this approach. The leakage flow has a constant flow rate R_leak_ and is driven by the concentration gradient between the ER and the cytosol.

$J_{Leak}=R_{leak} \left( {\left[ {Ca}^{2+} \right]_{ER}-\left[ {Ca}^{2+} \right]}_{Cyt} \right)$ (B17)

Unlike the other currents, it is not certain which cellular pathways are represented by the leak current, but its addition improves the results of the simulation, suggesting that a constant Ca^2+^ efflux from the ER is occuring. The backward flow of the second SERCA modeling approach was also tested by replacing the leakage term, but this did not yield the desired results.

### Diffusion between the junctions and the cytosol

Diffusion between the junctions and the cytosol can be described as a simple diffusion between two compartments through a solid membrane or barrier. In this case, the space between the inner junction region with the CRAC channels and the cytosol is considered as the diffusion barrier, as the concentration profile there decreases almost linearly towards the cytosol (Hogan, 2015, McIvor et al., 2018). With that arrangement, diffusion can be calculated using a standard linear diffusion equation:

$J_{Diff}=D_{j}*A_{diff}\frac{\left[ {Ca}^{2+} \right]_{Ju}-\left[ {Ca}^{2+} \right]_{Cyt}}{d_{diff}}$ (B18)

The diffusion distance d_diff_ is the thickness of the diffusion layer around the junction, A_diff_ is the lateral surface of the cylindrical junction and D_j_ is the diffusion coefficient for free intracellular calcium through the layer.

### Buffer Models

The binding of ions to buffer proteins can be described mathematically in different ways with varying degrees of accuracy. Buffer proteins often have several binding sites, which can have different binding affinities and capacities. Depending on the degree of accuracy, a mathematical model can describe the buffer with all different binding sites individually or with a single combined binding site (Means et al., 2006, Valeyev et al., 2008, Sala, and Hernández-Cruz, 1990). A simpler approach that can be used is the fast buffer approximation (Schmeitz et al., 2013, Lee and Keener, 2008). In this work, the buffer terms are described mathematically by a first order kinetic scheme according to (Sala, and Hernández-Cruz, 1990).

$\left[ {Ca}^{2+} \right]+\left[ B \right] \begin{matrix} \underset{\to}{k_{+}} \\ \overset{\leftarrow}{k_{-}} \end{matrix} \left[ CaB \right]$ (B19)

where [B] is the concentration of unbound buffer medium, [CaB] is the amount of Ca^2+^ bound buffer, and k_+_ and k_-_ are the forward and backward binding rates. The rate constants can be used to calculate the dissociation constant K_d_. The kinetic scheme can be written as an ODE, for which the following relationships are valid according to (Sala, and Hernández-Cruz, 1990):

$\frac{d\left[ B \right]}{dt}=k_{-}\left[ CaB \right]-k_{+}\left[ {Ca}^{2+} \right]\left[ B \right]$ (B20)

$\frac{d\left[ {Ca}^{2+} \right]}{dt}=\frac{d\left[ B \right]}{dt}$ (B21)

$\frac{d\left[ CaB \right]}{dt}=-\frac{d\left[ B \right]}{dt}$ (B22)

This basic concept is used for both buffer terms of the model. The cytosolic calcium buffer is described by a generic buffer term that does not represent a specific buffer molecule. Instead, the term [Ca^2+^]_b_ is introduced as a state variable describing the buffered cytosolic calcium. The concentration of the buffer agent [B] is replaced by the maximum possible buffered calcium [Ca^2+^]_b,tot_ minus the buffered cytosolic Ca^2+^ (Liu, 2012).

$\frac{d\left[ {Ca}^{2+} \right]_{Buff,Cyt}}{dt}=\frac{d\left[ {Ca}^{2+} \right]_{b}}{dt}={-k}_{off}\left[ {Ca}^{2+} \right]_{b}+k_{on}\left[ {Ca}^{2+} \right]_{cyt}\left( \left[ {Ca}^{2+} \right]_{b,tot}{-\left[ {Ca}^{2+} \right]}_{b} \right)$ (B23)

Here, k_off_ and k_on_ denote the backward and forward binding rates respectively, and [Ca^2+^]_b,tot_ can be interpreted as the maximum buffer capacity. This approach is a strong simplification of the different buffer molecules and their different binding sites (Means et al., 2006, Sala, and Hernández-Cruz, 1990), but the detailed modeling of these molecules would have the disadvantage of relying on the actual amount of buffer molecules in the cell. The initial concept of the model included a more detailed buffer term, but this did not improve the performance of the model due to the lack of reliable parameter estimates and was therefore replaced by the simplified aproach to save computational resources.

The second buffer term of the SOCE model represents the buffer calsequestrin (CSQ), which binds calcium in the ER. It is not the main calcium buffer in the ER of non-excitable cells and not crucial for the calcium dynamics, but it is a key agent for the gating of the RYR channel model shown in section “Model of the ryanodine receptor (RYR) channel”. The most important parameter for RYR channel gating is the concentration of unbound CSQ. The amount of calcium free calsequestrin [CSQ] corresponds to the variable [B] in the relationships of equation B30, and is thus given by (Lee and Keener, 2008, Sala, and Hernández-Cruz, 1990):

$\frac{d\left[ {Ca}^{2+} \right]_{Buff,ER}}{dt}=\frac{d\left[ CSQ \right]}{dt}=-k_{on,CSQ}\left[ CsQ \right]\left[ {Ca}^{2+} \right]_{ER}+k_{off,CSQ}\left( \left[ CSQ \right]_{total}-\left[ CSQ \right] \right)$ (B24)

Similar to equation B23, the constants k_on,CSQ_ and k_off,CSQ_ denote the forward and backward binding rates between Ca^2+^ and CSQ, and [CSQ]_total_ is the concentration of CSQ inside the ER.

### Model of the inositol 1,4,5-triphosphate (IP3) concentration

The gating of the IP3R channel model, which is explained in section “Model of the inositol (1, 4, 5) triphosphate receptor (IP3R) channel”, depends on the intracellular messenger IP3. The IP3 concentration can be modeled according to Liu, 2012:

$\frac{d\left[ IP3 \right]}{dt}=\frac{R_{ca}^{ip}\left[ {Ca}^{2+} \right]_{Cyt}}{K_{m}^{ca}+\left[ {Ca}^{2+} \right]_{Cyt}}+R_{d}^{ip}\left( \left[ \bar{IP3} \right]-\left[ IP3 \right] \right)$ (B25)

where $\left[ \bar{IP3} \right]$ is the maximum concentration of IP3. More sophisticated modeling approaches are available for the IP3 concentration (Diederichs, 2008), but this simplified description of IP3 is sufficient for the gating of the IP3R channel within the framework of this model.

# Part C

### Implementation of the SOCE model in MATLAB/Simulink

The mathematical model was implemented in MATLAB/Simulink (Version 2022b, Mathworks Inc.) and the system of equations solved with the forward Euler method (ODE1 in Simulink), with a fixed step size of 5*10^-7^s. The entire Simulink model is shown in **Figure C1**.


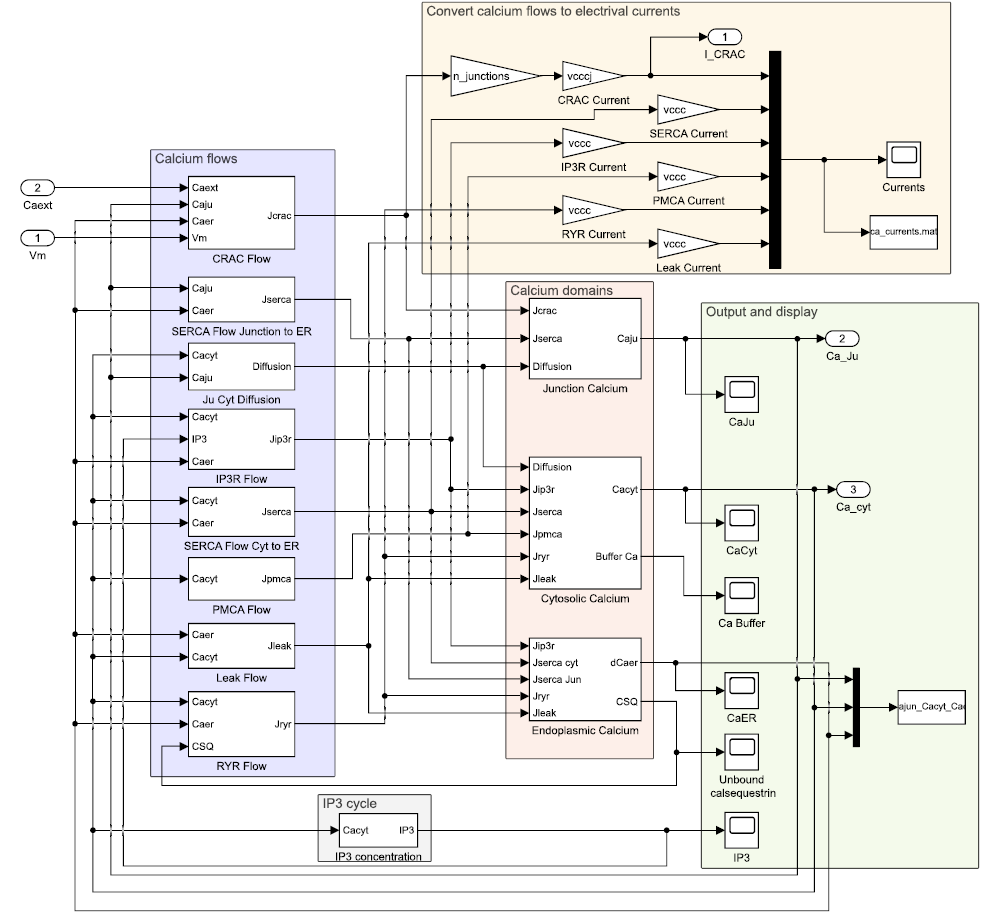


Supplementary Figure C1: Implementation of the SOCE model in Simulink. The blocks in the blue area represent the calcium flows. These are connected to the calcium domain blocks (red background). The buffer terms are contained in the respective calcium domain blocks. The yellow area contains the conversion of the ion flows into electrical currents via the vccc constant. The green area marks the outputs and display options. The IP3 concentration is included as a separate block.

Each calcium flow and calcium domain from the concept is embedded in its own Simulink block. The ion flows are held on the left and connected to the calcium domains on the right. The IP3 description is a separate block and, not included in the IP3R channel block. The buffer terms for the cytosol and the ER are both contained in their respective calcium domain blocks. The remaining Simulink blocks are required for converting the ion flows into electrical currents and for storing and displaying the simulation results. The constants and supporting functions used are defined in separate MATLAB scripts.

**Integration of the SOCE model into the A549 in-silico model**

The integration of the SOCE model into the A549 in-silico model was performed in MATLAB/Simulink (Version 2022b, Mathworks Inc). There, the SOCE model was masked into a single Simulink block and added to the existing Simulink version of the A549 in-silico model 1.0 (**Figure C2**). The equations are solved with the forward Euler method (ODE1 in Simulink), with a fixed step size of 5*10^-7^s.


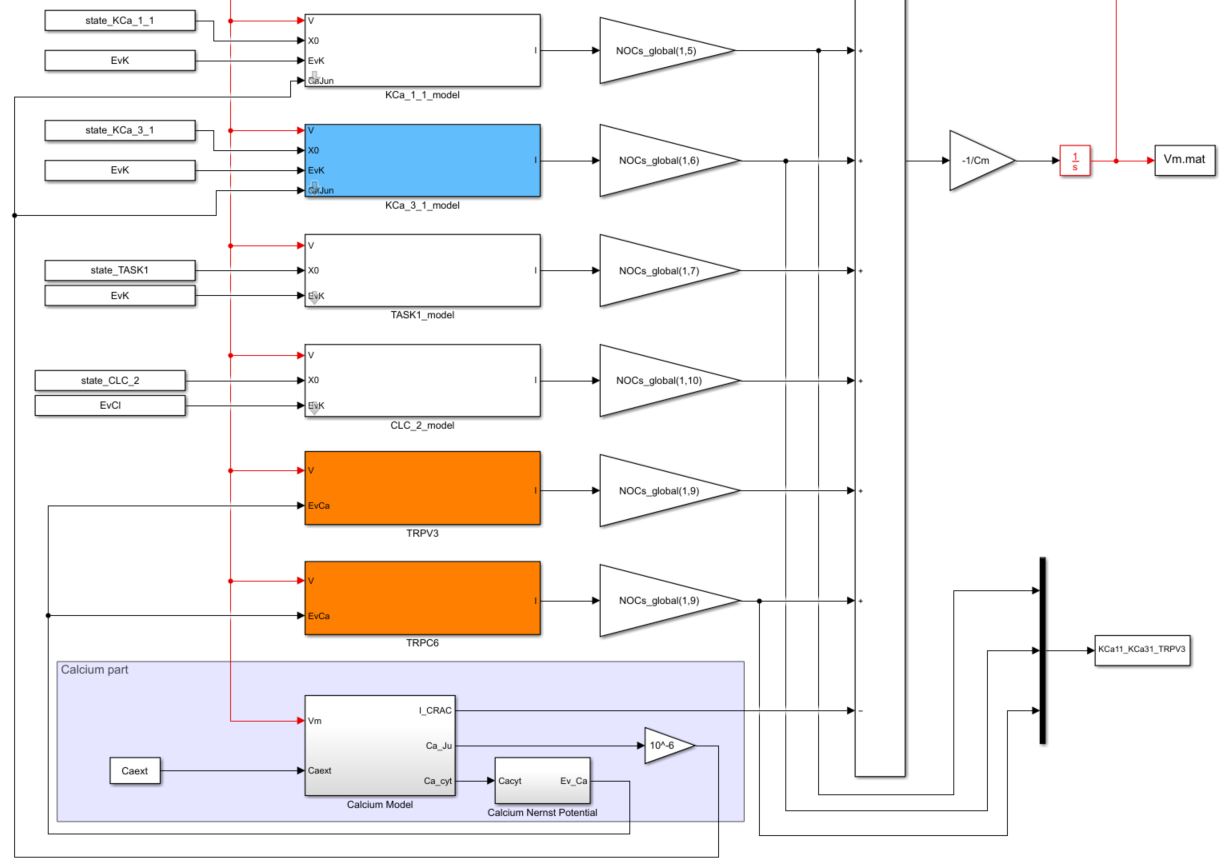


Supplementary Figure C2: Integration of the SOCE model into the A549 in Silico Simulink model. The block diagram shows the part of the original model that was modified. The blocks in the blue area are the new functional additions. The calcium model block contains the entire SOCE model

In general, the calcium submodel takes the membrane potential and [Ca^2+^]_Ext_ as inputs and gives [Ca^2+^]_Cyt_, [Ca^2+^]_Jun_ and the calcium current through all CRAC channels as output. The calcium concentration of the junction is used for the gating of the KCa channels, for which it needs to be scaled to match the dimensions of the initial A549 model. The cytosolic calcium is used to calculate the calcium Nernst potential for the TRPV3 and TRPC6 channels, which is implemented in a separate “block” within the A549 model. The CRAC current is used to calculate the whole cell current with a negative sign, as the SOCE model accounts for all unsigned calcium currents and the voltage model requires signed currents. All required parameter settings are previously loaded to the Simulink workspace using a separate MATLAB script.

# Part D

## Variation of the model parameters

The simulations in **Figure D1 a-c** are shown for the cytosolic calcium concentration. Each sub-figure yields a variation of a single parameter, while blue lines serve as the reference simulation with the basic settings used for **Figure 4.** The parameters were chosen so that they remain within reasonable limits.

**Figure D1 a** shows the parameters that affect the maximum calcium concentration during the calcium entry through the CRAC channels. Increasing the number of junctions, as shown in the top panel, directly increases the number of CRAC channels and therefore the cytosolic calcium during the store-operated calcium entry (SOCE). The length of the diffusion distance appears to have an inverse effect on [Ca^2+^]_Cyt_. The different PMCA activity scales the cytosolic calcium throughout the entire experiment, but does not change the shape of the curve. The effects of variations at a single junction are shown in **Figure D1 b**. The number of junctions and the PMCA activity have a similar effect on the junction as on the cytosol, which is not clearly visible on the scale of **Figure D1 b.** They cause only a slight change on this scale compared to the diffusion distance, which influences [Ca^2+^]_Jun_ considerably.

The impact of the buffer capacities on [Ca^2+^]_Cyt_ is shown in **Figure D1 c**. The top panel shows the variation of the cytosolic buffer capacity. At a buffering capacity of 66 µM calcium, [Ca^2+^]_Cyt_ exhibits a sharp spike during calcium release from the ER with a higher peak value and steeper slopes during the SOCE phase. A higher buffer capacity smoothes the slopes and slightly raises the calcium baseline. Of all the parameters varied, the buffer capacity is the only one that has an effect on the calcium drop at the end of the simulation. CSQ mainly effects [Ca^2+^]_ER_, but also influences [Ca^2+^]_Cyt_ as can be seen in the middle panel of **Figure D1 c**. The difference between 14 µM and 140 µM is very small, but at 1400 µM CSQ, [Ca^2+^]_Cyt_ is significantly greater throughout the ER outflow. The bottom panel shows the variation of the maximal SERCA pump rate, with resting calcium decreasing as SERCA activity increases.


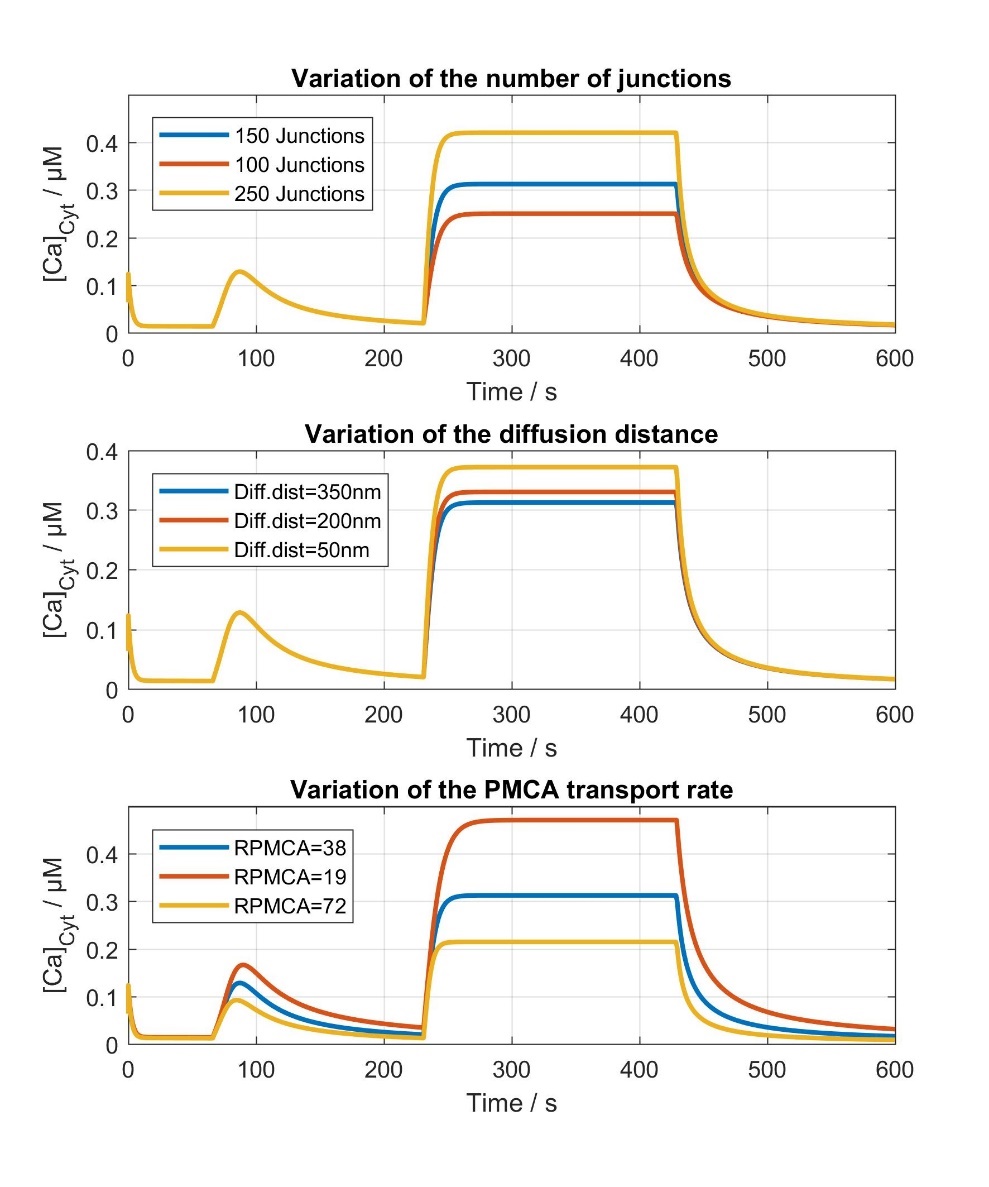


Supplementary Figure D1 a: Cytosolic calcium concentrations with different parameter settings of the SOCE model. Top: Variation of the number of ER-PM junctions. Mid: Variation of the diffusion distance between the ER-PM junction and the cytosol. Bottom: Variation of the PMCA pump transport rate with unit µM/s.


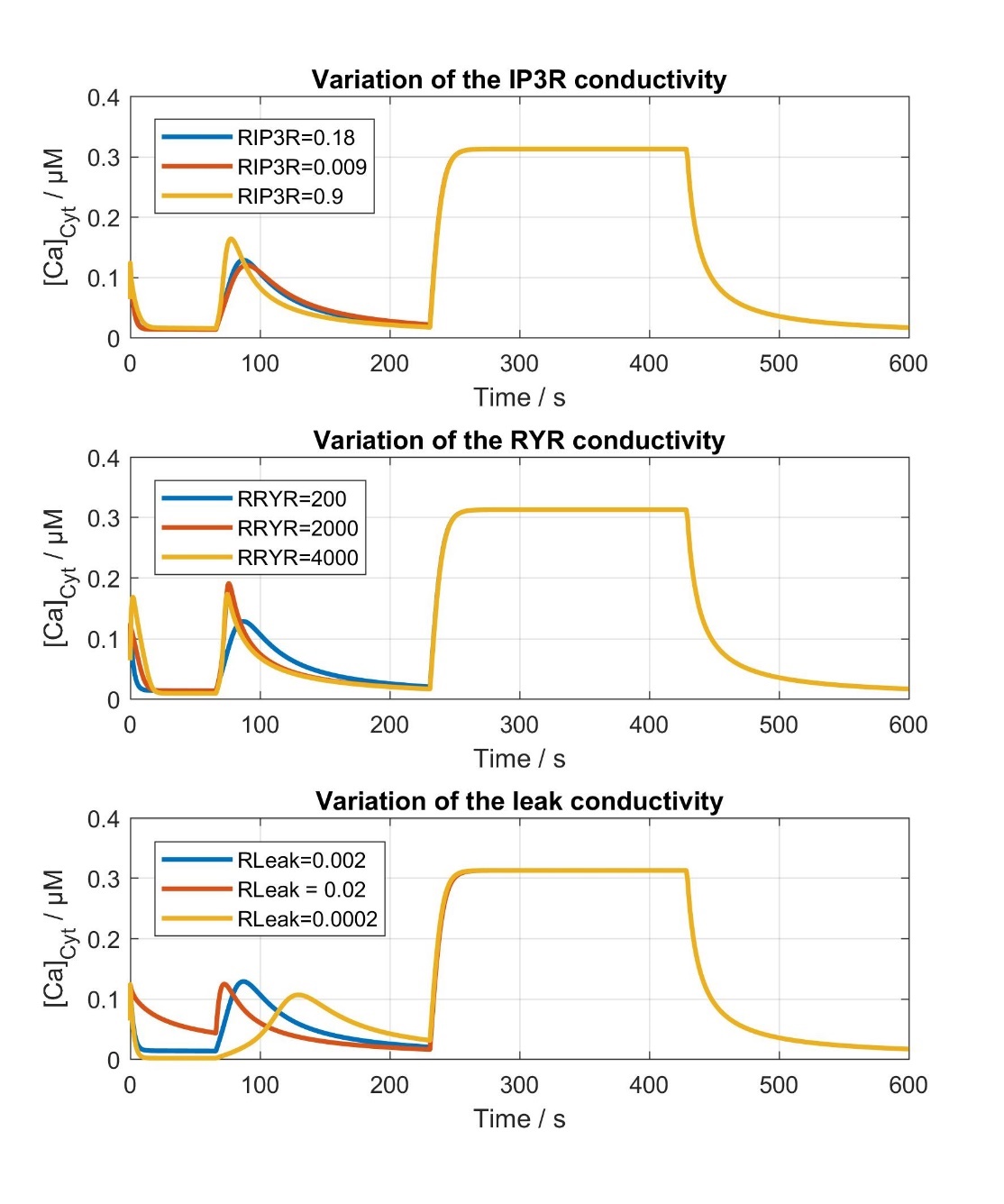


Supplementary Figure D1 b: Cytosolic calcium with different settings during the SOCE simulation. Top: Variation of the IP3R channel conductivity or transfer rate with the unit µM/s. Middle: Variation of the RYR channel transport rate in µM/s. Bottom: Variation of the ER leakage rate in µM/s


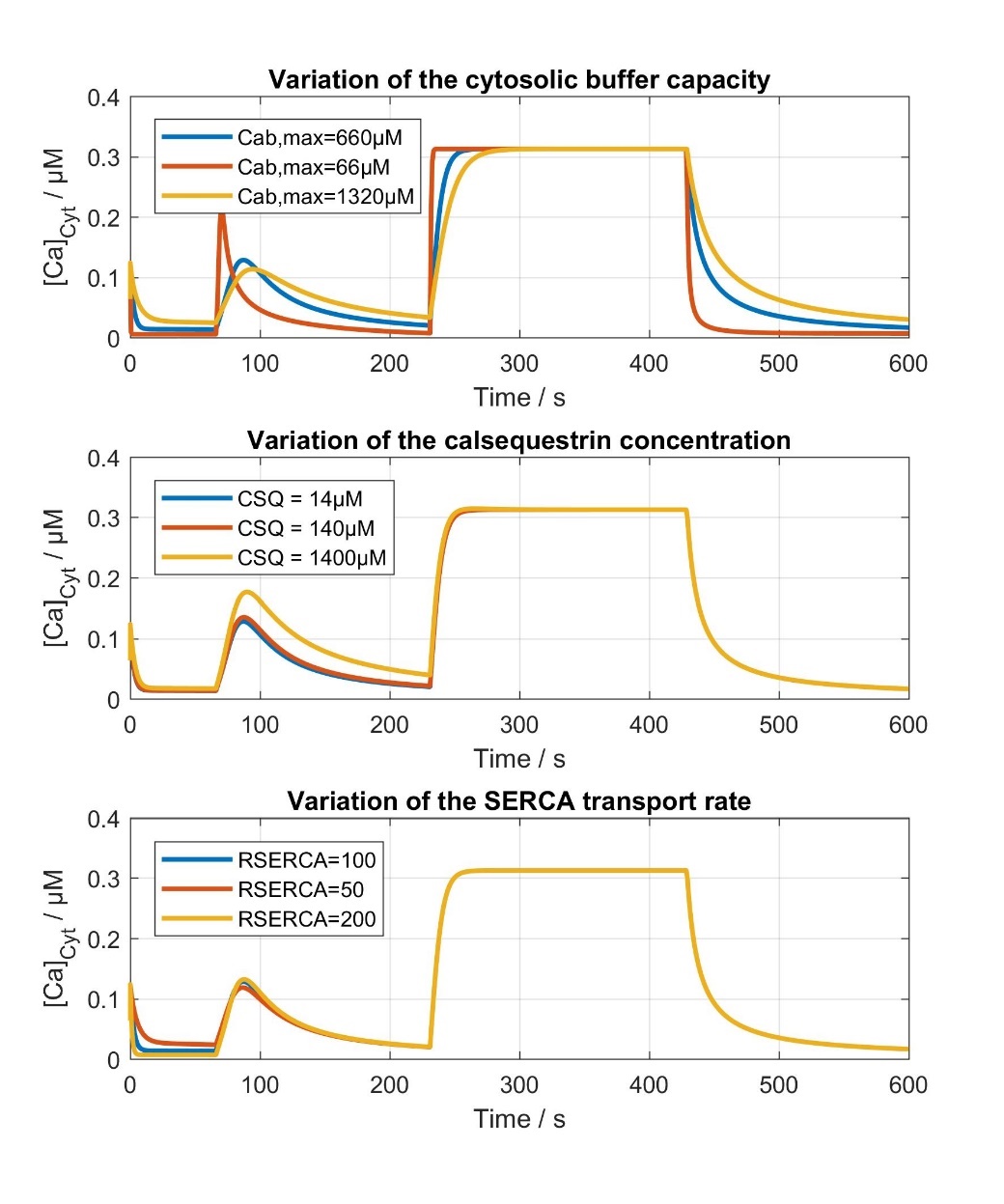


Supplementary Figure D1 c: Cytosolic calcium with different settings during the SOCE simulation. Top: Variation of the maximal amount of buffered calcium in the cytosol. Mid: Variation of the amount of calsequestrin in the ER. Bottom: Variation of the SERCA pump transport rate with unit µM/s

**References**

Berridge, M., Lipp, P. & Bootman, M. The versatility and universality of calcium signalling. *Nat Rev Mol Cell Biol* **1**, 11–21 (2000). https://doi.org/10.1038/35036035

Chemaly, E.R., Troncone, L., Lebeche, D. (2018) SERCA control of cell death and survival. Cell. Calcium. 69, 46–61. doi: 10.1016/j.ceca.2017.07.001

Chen, Y.W., Chen, Y.F., Chen, Y.T., Chiu, W.T., Shen, M.R. (2016) The STIM1-Orai1 pathway of store-operated Ca2+ entry controls the checkpoint in cell cycle G1/S transition. Sci. Rep. 6, 22142. doi: 10.1038/srep22142

Clapham, D.E. (2003) TRP channels as cellular sensors. Nature. 426(6966), 517–24. DOI: 10.1038/nature02196

Diederichs, F. (2008) Ion homeostasis and the functional roles of SERCA reactions in stimulus-secretion coupling of the pancreatic beta-cell: A mathematical simulation. Biophys. Chem. 134(3), 119–143. doi: 10.1016/j.bpc.2008.02.001

EMBL-EBI. (2023) Expression Atlas. https://www.ebi.ac.uk/gxa/search?geneQuery=%5B%7B%22value%22%3A%22STIM1%22%7D%5D&species=homo%20sapiens&conditionQuery=%5B%7B%22value%22%3A%22A549%22%7D%5D&bs=%7B%22homo%20sapiens%22%3A%5B%22CELL_LINE%22%5D%7D&ds=%7B%22kingdom%22%3A%5B%22animals%22%5D%7D#baseline

Estacion, M., Sinkins, W.G., Jones, S.W., Applegate, M.A., Schilling, W.P. (2006) Human TRPC6 expressed in HEK 293 cells forms non-selective cation channels with limited Ca2+ permeability. J. Physiol. 572(Pt 2), 359-77. doi: 10.1113/jphysiol.2005.103143.

Hogan, P.G. (2015) The STIM1-ORAI1 microdomain. Cell Calcium. 58(4), 357–367. doi: 10.1016/j.ceca.2015.07.001

Hou, M.F., Kuo, H.C., Li, J.H., Wang, Y.S., Chang, C.C., Chen, K.C. et al. (2011) Orai1/CRACM1 overexpression suppresses cell proliferation via attenuation of the store-operated calcium influx-mediated signalling pathway in A549 lung cancer cells. Biochim. Biophys. Acta. 1810(12), 1278–1284. doi: 10.1016/j.bbagen.2011.07.001

Islam, Md.S. (2020) Calcium Signaling. Springer Cham. doi.org/10.1007/978-3-030-12457-1

Khan, H.Y., Mazahir, I., Reddy, S., Fazili, F., Azmi, A. (2020) Roles of CRAC channel in cancer: implications for therapeutic development. Expert Rev. Precis. Med. Drug. Dev. 5(5), 371–382. doi: 10.1080/23808993.2020.1803062

Kowalewski, J.M., Uhlén, P., Kitano, H., Brismar, H. (2006) Modeling the impact of store-operated Ca2+ entry on intracellular Ca2+ oscillations. Math. Biosci. 204(2), 232–49. doi: 10.1016/j.mbs.2006.03.001

Kozak, J.A., Putney, J.W. (2018) Calcium Entry Channels in Non-Excitable Cells. Boca Raton (FL): CRC Press/Taylor & Francis. doi: 10.1201/9781315152592

Lanner, J.T., Georgiou, D.K., Joshi, A.D., Hamilton, S.L. (2010) Ryanodine receptors: structure, expression, molecular details, and function in calcium release. Cold Spring Harb. Perspect. Biol. 2(11), a003996. doi: 10.1101/cshperspect.a003996

Lee, Y.S., Keener, J.P. (2008) A calcium-induced calcium release mechanism mediated by calsequestrin. J. Theor. Biol. 253(4), 668–679. doi 10.1016/j.jtbi.2008.04.027

Liu, W., Tang, F., Chen, J. (2010) Designing dynamical output feedback controllers for store-operated Ca^2^+ entry. Math. Biosci. 228(1), 110–118. doi: 10.1016/j.mbs.2010.08.013

Liu, W. (2012) Introduction to Modeling Biological Cellular Control Systems. Springer Milano. doi: 10.1007/978-88-470-2490-8

Liu, W. (2012) “Store-Operated Calcium Entry” in Introduction to Modeling Biological Cellular Control Systems, ed W. Lui. Springer Milano. pp. 189–206. doi: 10.1007/978-88-470-2490-8_8

Lopez, J.J., Jardin, I., Sanchez-Collado, J., Salido, G.M., Smani, T., Rosado, J.A. (2020) TRPC Channels in the SOCE Scenario. Cells. 9(1), 126. doi: 10.3390/cells9010126

Lytton, J., Westlin, M., Burk, S.E., Shull, G.E., MacLennan, D.H. (1992) Functional comparisons between isoforms of the sarcoplasmic or endoplasmic reticulum family of calcium pumps. J. Biol. Chem. 267(20), 14483–14489.

McIvor, E., Coombes, S., Thul, R. (2018) Three-dimensional spatio-temporal modelling of store operated Ca2+ entry: Insights into ER refilling and the spatial signature of Ca2+ signals. Cell. Calcium. 73, 11–24. doi: 10.1016/j.ceca.2018.03.006

Means, S., Smith, A.J., Shepherd, J., Shadid, J., Fowler, J., Wojcikiewicz, R.J.H., et al. (2006) Reaction diffusion modeling of calcium dynamics with realistic ER geometry. Biophys. J. 91(2), 537–557. doi: 10.1529/biophysj.105.075036

Moccia, F. (2018) Endothelial Ca2+ Signaling and the Resistance to Anticancer Treatments: Partners in Crime. Int. J. Mol. Sci. 19(1), 217. doi: 10.3390/ijms19010217

Owsianik, G., Talavera, K., Voets, T., Nilius, B. (2006) Permeation and selectivity of TRP channels. Annu. Rev. Physiol. 68, 685–717. doi: 10.1146/annurev.physiol.68.040204.101406

Padar, S., van Breemen, C., Thomas, D.W., Uchizono, J.A., Livesey, J.C., Rahimian, R. (2004) Differential regulation of calcium homeostasis in adenocarcinoma cell line A549 and its Taxol-resistant subclone. Br. J. Pharmacol. 142(2), 305–316. doi: 10.1038/sj.bjp.0705755

Peters, A.A., Milevskiy, M.J.G., Lee, W.C., Curry, M.C., Smart, C.E., Saunus, J.M., et al. (2016) The calcium pump plasma membrane Ca(2+)-ATPase 2 (PMCA2) regulates breast cancer cell proliferation and sensitivity to doxorubicin. Sci. Rep. 6, 25505. doi: 10.1038/srep25505

Rezuchova, I., Hudecova, S., Soltysova, A., Matuskova, M., Durinikova, E., Chovancova, B., et al. (2019) Type 3 inositol 1,4,5-trisphosphate receptor has antiapoptotic and proliferative role in cancer cells. Cell Death Dis. 10(3),186. doi: 10.1038/s41419-019-1433-4

Roberts-Thomson, S.J., Curry, M.C., Monteith, G.R. (2010) Plasma membrane calcium pumps and their emerging roles in cancer. World J. Biol. Chem. 1(8), 248–253. doi: 10.4331/wjbc.v1.i8.248

Sala, F., Hernández-Cruz, A. (1990) Calcium diffusion modeling in a spherical neuron. Relevance of buffering properties. Biophys. J. 57(2), 313–24. doi: 10.1016/S0006-3495(90)82533-9

Schmeitz, C., Hernandez-Vargas, E.A., Fliegert, R., Guse, A.H., Meyer-Hermann, M. (2013) A mathematical model of T lymphocyte calcium dynamics derived from single transmembrane protein properties. Front. Immunol. 4, 277. doi: 10.3389/fimmu.2013.00277

Shin, D.H., Leem, D.G., Shin, J.S., Kim, J.I., Kim, K.T., Choi, S.Y. et al. (2018) Compound K induced apoptosis via endoplasmic reticulum Ca2+ release through ryanodine receptor in human lung cancer cells. J. Ginseng. Res. 42(2), 165–174. doi: 10.1016/j.jgr.2017.01.015

Sneyd, J., Dufour, J.F. (2002) A dynamic model of the type-2 inositol trisphosphate receptor. Proc. Natl. Acad. Sci. U. S. A. 99(4), 2398–2403. doi: 10.1073/pnas.032281999

Tatur, S., Groulx, N., Orlov, S.N., Grygorczyk, R. (2007) Ca2+-dependent ATP release from A549 cells involves synergistic autocrine stimulation by coreleased uridine nucleotides. J. Physiol. 584(Pt 2), 419–35. doi: 10.1113/jphysiol.2007.133314

Thomas, R.C. (2009) The plasma membrane calcium ATPase (PMCA) of neurones is electroneutral and exchanges 2 H+ for each Ca2+ or Ba2+ ion extruded. J. Physiol. 587(2), 315–27. doi: 10.1113/jphysiol.2008.162453

Valeyev, N.V., Bates, D.G., Heslop-Harrison, P., Postlethwaite, I., Kotov, N.V. (2008) Elucidating the mechanisms of cooperative calcium-calmodulin interactions: a structural systems biology approach. BMC Syst. Biol. 2, 48. doi: 10.1186/1752-0509-2-48

Wang, F., Liu, D-zhong., Xu, H., Li, Y., Wang, W., Liu, L-you., et al. (2014) Thapsigargin induces apoptosis by impairing cytoskeleton dynamics in human lung adenocarcinoma cells. ScientificWorldJournal. 2014, 619050. doi: 10.1155/2014/619050

Xu, H., Ramsey, I.S., Kotecha, S.A., Moran, M.M., Chong, J.A., Lawson, D., et al. (2002) TRPV3 is a calcium-permeable temperature-sensitive cation channel. Nature. 418(6894), 181–186. doi: 10.1038/nature00882
